# Transport Properties of Melanosomes along Microtubules Interpreted by a Tug-of-War Model with Loose Mechanical Coupling

**DOI:** 10.1371/journal.pone.0043599

**Published:** 2012-08-30

**Authors:** Sebastián Bouzat, Valeria Levi, Luciana Bruno

**Affiliations:** 1 Centro Atómico Bariloche - Comisión Nacional de Energía Atómica, Bariloche, Río Negro, Argentina; 2 Departamento de Química Biológica, Facultad de Ciencias Exactas y Naturales, Universidad de Buenos Aires, Ciudad de Buenos Aires, Buenos Aires, Argentina; 3 Departamento de Física, Facultad de Ciencias Exactas y Naturales, Universidad de Buenos Aires, Ciudad de Buenos Aires, Buenos Aires, Argentina; Institut Curie, France

## Abstract

In this work, we explored theoretically the transport of organelles driven along microtubules by molecular motors of opposed polarities using a stochastic model that considers a Langevin dynamics for the cargo, independent cargo-motor linkers and stepping motion for the motors. It has been recently proposed that the stiffness of the motor plays an important role when multiple motors collectively transport a cargo. Therefore, we considered in our model the recently reported values for the stiffness of the cargo-motor linker determined in living cells (∼0.01 pN/nm, [1]) which is significantly lower than the motor stiffness obtained in *in vitro* assays and used in previous studies. Our model could reproduce the multimodal velocity distributions and typical trajectory characteristics including the properties of the reversions in the overall direction of motion observed during melanosome transport along microtubules in *Xenopus laevis* melanophores. Moreover, we explored the contribution of the different motility states of the cargo-motor system to the different modes of the velocity distributions and could identify the microscopic mechanisms of transport leading to trajectories compatible with those observed in living cells. Finally, by changing the attachment and detachment rates, the model could reproduce the different velocity distributions observed during melanosome transport along microtubules in *Xenopus laevis* melanophores stimulated for aggregation and dispersion. Our analysis suggests that active tug-of-war processes with loose mechanical coupling can account for several aspects of cargo transport along microtubules in living cells.

## Introduction

Transport along microtubules of a wide variety of cellular cargoes is driven by the molecular motors kinesin and dynein which perform processive steps toward the plus and minus ends of the microtubules using energy provided by ATP hydrolysis.

A common observation during microtubule-dependent transport in living cells is that cargoes frequently reverse their main direction of motion and move back and forth toward the minus and plus ends (see for example, [2], [3]). Examples of this bidirectional motion have been reported for lipid droplets transport in *Drosophila* embryos [4], axonal transport [5], [6], [7] and organelle transport in different cellular systems [8], [9], [10]. Despite the relevance of this process, the mechanisms underlying bidirectional transport remain unclear.

To explore this process, several research groups have followed the motion of different cargoes in living cells using a variety of single particle tracking techniques. These trajectories are quantitatively analyzed to determine properties of the transport process such as run lengths and pauses durations [11], [12].

One of key parameters determined in these analyses is the segmental velocity, which is obtained in regions of the trajectories where the cargo moves unidirectionally at a constant velocity [8]. It has been shown that the histogram of segmental velocities presents a multimode distribution in different cellular systems [6], [8], [13]. This behavior has been qualitatively described considering that high-velocity peaks are due to the joint action of multiple copies of a given motor molecule [8], [13]. This initial and relatively simple cooperative model in which motors of opposed polarities were considered inactive during the transport has been changed and improved over the years due to new experimental and theoretical evidences pointing toward models that also consider active tug of war between microtubule motors during transport [14], [15], [16], [17]. However, the molecular mechanisms involved in the transport driven by multiple copies of motor molecules of identical or opposed polarity are still a subject of debate in the field [18].

In order to explore the molecular mechanisms involved in multimotor driven transport, several groups have also assay the properties of tandems of motors in *in vitro* conditions [14], [19], [20], [21]. Particularly, Bieling et al. [20] have determined that stiff constructs of kinesin-1 lacking most of the non-motor and potentially flexible regions interfere with each other when collectively transporting a cargo demonstrating that a high elasticity is essential for a loose mechanical coupling between motors.

Bidirectional transport along microtubules have been explained by two different models: the mean-field models which assumes that all engaged motors of the same polarity share the load equally [16], and the stochastic models which considers unequal load sharing among the motors [17], [18], [22], [23], [24], [25], [26]. Theoretical and experimental data suggest that stochastic models provide better descriptions of the transport processes [17], [18], [24]. While stochastic models predict some of the features of organelle transport, it has not been explored if they are able to reproduce the multimodal distribution of segmental velocities previously mentioned.

Numerical modeling of multimotor transport requires inputting values of motor parameters such as stall force, attachment and detachment rates from the microtubule and motor stiffness. This last parameter -which has been shown to play a key role for the mechanical coupling of motors [20]- is generally considered to be ∼0.3 pN nm, value that corresponds to the stiffness of conventional kinesin determined in *in vitro* conditions [27], [28], [29].

In living cells, other biomolecules involved in the interaction between motors and organelles could also contribute to the mechanical properties of the motor linker. In this way, it has been showed that the stiffness of the complex constituted by active microtubule motors and molecules mediating their attachment to melanosomes in *Xenopus laevis* melanophores is ∼0.03 pN nm, which is one order lower than that reported for kinesin in *in vitro* conditions [1].

Regarding the values of the stall forces of motors, there is some controversy in the literature. In the case of dynein, different *in vitro* experiments report values ∼1 pN [30], [31], [32], while other asserts values ∼7 pN [33], [34]. Furthermore, values around 2.5 pN [35] and in the range 3–5 pN [36] have been reported for single dyneins in living cells.

Another important topic refers to the configuration of motors driving the cargoes. Strong asymmetry between kinesin and dynein motors regarding stall forces and number of active motors have been reported *in vitro* [30], [31], [32] and in endosome transport in *Dictyostelium* cells [10], while in other cellular systems, such as *Drosophila* embryos, other authors assert symmetric behaviors for kinesins and dyneins [18], [35], [36].

The discrepancies described above might be originated in the difficulty on determining the number of motors driving an organelle in living cells. Typically, these measurements are based on the analysis of the run lengths distributions and their comparison with the distributions obtained *in vitro*. However, it has been demonstrated (see, for example, [18], [35]) that these quantities (run lengths and stall forces) can be different *in vivo* and *in vitro*.

In this work, we propose a stochastic tug-of-war model [23], [37] to describe the bidirectional transport of melanosomes in *Xenopus laevis* melanophores, which takes into account the low stiffness values found in the experiments. We compare the predictions of the model with previous experimental results [8], [38] and observe that the model allows recovering the overall characteristics of the experimental trajectories and, in particular, the reversions on the transport direction. Moreover, the model also predicts a multimodal distribution for segmental velocities as observed in living cells. In view of the prevailing controversy on the stall forces of motors, in our model we consider values from 1 pN to 6 pN for this parameter.

Since the number of dynein and kinesin motors participating in the transport of melanosomes in *Xenopus laevis* melanophores is unknown as far as we know, we deal mainly with symmetric arrays since this reduces considerably the number of free parameters of the model. However, we will also consider the case of strong asymmetric configurations of motors.

Regardless the different parameters values considered, our results suggest that active tug-of-war processes with loose mechanical coupling can account for several aspects of cargo transport along microtubules in living cells.

## Model and Numerical Simulations

In this work we consider a slightly modified version of the model for cargo transport by two opposing motor teams proposed in [23], [24]. The model shares three main assumptions with other one-dimensional models proposed in the literature [17], [22]: a continuous dynamics for the cargo along a spatial coordinate *x* representing the position on the microtubule (MT), a stochastic stepping dynamics for the motors in the same coordinate, and independent elastic linkers for individual motors.

**Figure 1:**
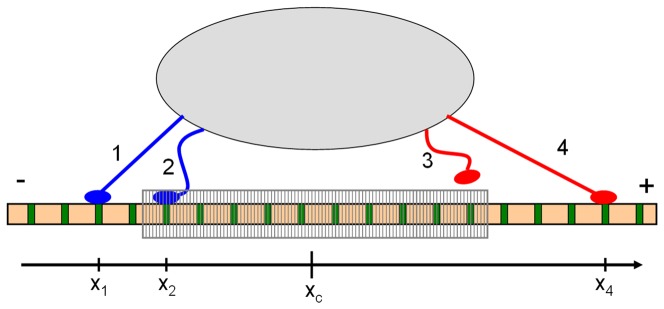
Scheme of the model. Example of an instantaneous configuration of the system with *N_f_ = N_b_ = *2. The cargo is represented by a grey bubble. Forward motors (kinesin) are represented in red, while backward motors (dynein) in blue. The MT has green sites separated by Δx = 8 nm where motors can engage and step. The shaded zone around the cargo position indicates the region of the MT where attached motors do not exert forces on the cargo. Motors are labeled with numbers from 1 to 4. From left to right the figure shows a backward pulling motor (labeled as 1), a backward non-pulling motor (2), a detached forward motor (3) and a forward pulling motor (4). The attachment state (see main text) is thus (*n_f_,n_b_*) = (1,2), while the pulling state is (*q_f_,q_b_*) = (1,1). Although *q_f_ = q_b_*, the force on motor 4 is larger than that exerted on motor 1 since |x_4_−x_c_|>|x_1_−x_c_| (see Eq. 1). Thus, the cargo moves to the right.

In Figure 1 we show a schematic cartoon of the system. The cargo (represented by the gray bubble in Fig. 1) is assumed to be permanently linked to *N_f_* forward motors and *N_b_* backward motors. We consider each motor as constituted by a point-like domain, which binds to the MT, plus an elastic element that connects it to the cargo. In what follows, we will use the words *motor* and *motor-linker* to refer to the point-like domain and the elastic linker, respectively. At a given time *t*, each motor may be either attached to the MT or detached. The allowed positions for engaged motors are the discrete sites *x_j_* = *jΔx*, with integer *j* and *Δx = *8* nm*, represented with green bars in Figure 1. We call *n_f_(t)* and *n_b_(t)* the number of engaged forward and backward motors, respectively. In the configuration shown in Figure 1 we have *N_f_* = *N_b_* = 2, *n_f_(t)* = 1 and *n_b_(t)* = 2 (see caption for further indications).

Following references [17], [22], [23], [24], the elastic properties of the motor-linkers are considered such that only the motors located beyond a critical distance *x_0_* from the cargo exert forces. The forces are always attractive. The motors engaged beyond that zone (shaded region in Fig. 1) are called *pulling motors*. The number of forward and backward pulling motors are denoted as *q_f_(t)* and *q_b_(t)*, respectively. In the instantaneous configuration shown in Figure 1 we have *q_f_ (t) = q_b_(t) = *1, meaning that there is a tug of war between a forward motor and a backward one. The figure also shows that the motor-linker of the pulling forward motor is more extended than that of the backward pulling motor. Thus, in this particular condition the cargo would move toward the plus end of the MT. The distinction between attached motors and pulling motors is relevant; for instance, the number of attached motors is related to the run lengths of the cargo motion, while the pulling motors control the cargo velocity [23], [24], [26].

Let us now introduce the detailed formulation of the model. We call *x_c_(t)* the cargo position as a function of time, and *x_i_(t)* with *i = *1*…N_b_+N_f_*, the position of the motors along the microtubule. For an attached motor *i*, we define *d_i_ = x_i_(t)−x_c_(t)*, the distance between the motor and the cargo. Then, the instantaneous force *f_i_* that motor *i* exerts on the cargo is defined as:
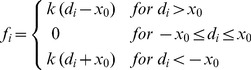
(1)Here, *k* is the stiffness of an individual motor-linker. This parameter models the effective resistance against stretching of the molecular motor and its adaptors, considering all the different structures between the MT and the organelle.

From Eq. 1 it is clear that the pulling motors can be formally defined as those attached outside the region |*x_i_(t)−x_c_(t)*|*<x_0_*, which corresponds to the shaded region shown in Figure 1. Throughout this work we consider *x_0_* = 110 nm [17], [22], [23], [24].

The cargo position evolves according to the Langevin equation:
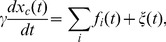
(2)where *γ* is the viscous drag, *f_i_* is the force exerted by the i-th motor on the cargo (Eq. 1), and ξ is white thermal noise [22], [23]. The thermal noise intensity is K_B_T/*γ*, with K_B_ the Boltzmann constant and T the temperature (equal to 300 K in the following sections), satisfying the fluctuation-dissipation relation as explained in [24]. The viscous drag follows the behavior predicted by the Stokes relation *γ = 6πη*r, where *η* is the viscosity of the medium and r the cargo radius. For convenience, we define the dimensionless viscosity n_γ_ = η/η_o_ -with η_o_ the viscosity of water-, which will be used as the control parameter for the viscosity.

Concerning the motion of the attached motors, the corresponding stepping dynamics is ruled by the loading forces *L_i_* acting on the motors. These loading forces are the mechanical reactions to the forces *f_i_* defined by Eq. 1. We consider *L_i_*>0 when exerted against the polarity of the motor, so we have *L_i_ = f_i_* for forward motors and *L_i_ = −f_i_* for backward ones.

According to the model, each attached motor *i* performs a step of length *Δx* = 8 nm towards the direction determined by its polarity with a probability per time unit equal to *p_step_* (*L_i_*) = *v_i_*(*L_i_*)/*Δx*. For *v_i_*(*L_i_*), the mean velocity of the motor as a function of the load, we assume the following expression [16], [17], [22], [23], [24]:
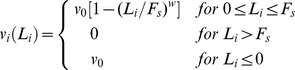
(3)where *v_0_*>0 is the motor velocity at zero load, *F_s_*>0 is the stall force and *w>0* is an exponent of nonlinearity. We consider parameters *v_0f_*, F*_sf_*, *w_f_* for forward motors and *v_0b_*, F*_sb_*, *w_b_* for backward motors. Note that, in contrast with the assumptions in [23], no back steps are allowed. This simplification reduces the number of the system parameters without changing the results in a significant way [23].

In order to describe completely the stepping dynamics, attachment and detachment rules must be defined. On the one hand, every detached motor engages to the microtubule with rate *π*_f_ or *π*_b_, depending on its type. The attachment occurs with equal probability at any of the discrete sites *x_j_* belonging to the region |*x_j_*−*x(t)*|<*x_0_*, which means that during the attachment process the motor-linker is relaxed. On the other hand, each attached motor *i* detaches from the microtubule with a probability per time unit given by [17], [22]:
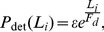
(4)where ε is the zero-load detachment rate and *F_d_* is the detachment force of the motor. We named ε_f_, ε_b_, *F_df_*, and *F_db_*, the corresponding parameters for forward and backward motors.

The exponential dependence of *P_det_* on the load (Eq. 4) is a usual assumption for the detachment rate in many models for transport by multiple motors [17], [18], [22], [23], [24], [25]. It provides a simple characterization of the detachment processes inspired by *in vitro* experiments with kinesin [39] and by the Kramers' theory for barrier crossing [40]. In the present work we assume such a simple formulation since there is no detailed knowledge of the detachment rates for kinesin 2 and dynein in melanophores. Thus, we consider ε_f_, ε_b_, *F_df_*, and *F_db_* as parameters to be fitted.

Given a dynamical model with a large number of parameters it is always desirable to find a combination of parameter transformations that leaves the dynamics invariant. This reduces the effective dimensionality of the parameter space and, thus, facilitates the analysis [40]. Interestingly, the simulations of the model presented here show that the transformation *F_s_*→λ*F_s_ F_d_*→β*F_d_, k*→λ*k* and γ→λγ, with 0.4<λ<1 a dimensionless parameter and β a number of order λ^1/2^, lead to a scaled system whose dynamical properties are similar to those of the original one (see Information S2). We will use this property to study the behavior of our model for different values of the parameters, particularly concerning the results for varying stall forces.

Numerical implementations of the model requires a 10^−5^–10^−7^ s discretization of time, depending on *γ*. As initial conditions we consider that all the motors are engaged. The simulation lasts 20 s or until all the motors are detached.

### Attachment and pulling states

We call the *attachment state* of the system to (*n_f_*(*t*), *n_b_*(*t*)), where *n_f_*(*t*) and *n_b_*(*t*) are the number of attached forward and backward motors at a given time.

Similarly, we define the *pulling state* as (*q_f_*(*t*), *q_b_*(*t*)), with the information of the pulling motors. In the particular configuration shown in Figure 1, the attachment state is (*n_f_*, *n_b_*) = (1,2), while the pulling state is (*q_f_*, *q_b_*) = (1,1).

### Simulated trajectories, segmental velocities and velocity distributions

**Figure 2:**
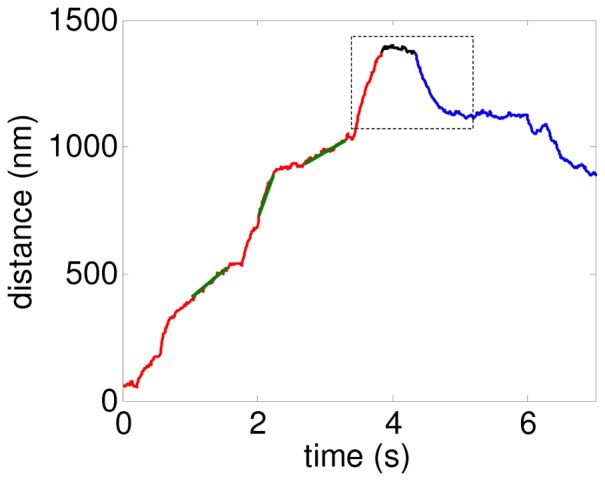
Trajectories and segments. Representative trajectory of a cargo obtained during a simulation of the model with *N_f_* = *N_b_* = 2 showing a processive trend in the forward direction (red trace) followed by a reversion (dashed rectangle) and, finally, a motion in the backward direction (blue trace). Segmental velocities are determined from pieces of the trajectory containing 40 data points during which the cargo moves with constant velocity, such as the ones indicated with green traces. The system is symmetric and the corresponding parameters are displayed in Table 1.

Figure 2 shows an example of a simulated trajectory for a cargo driven by two forward and two backward motors. Clearly, we can observe a period of processive motion towards the plus end of the MT (plotted in red) followed by a period of motion towards the minus end (in blue). Short periods of constant local velocity (indicated in green) can also be distinguished. In this particular trajectory, we can also see that the reversion pattern during the change from forward to backward motion is wave shaped, as defined in [38].

In order to determine the local velocities of the cargo, we followed the procedure used by Levi et al. [8]. In brief, we sampled the trajectories with a time step of 0.01 s, i.e. at least 1000 times larger than the computational time step. Then, we selected periods of processive motion using the method described in Information S1. After this, each selected period was further divided into segments of 40 points (i.e. 0.4 s) and the mean velocity of the segment was determined as the slope of the position vs. time plot. Finally, we built the histograms for the segmental velocities in the plus and minus direction using the same criterion as in [8] for defining the bin size. We only included in the histograms segments with speeds >100 nm/s and with correlation coefficient for the linear fitting higher than 0.98 [8]. For each set of parameters we typically analyzed 1000–4000 trajectories.

Note that each 40-point segment with constant velocity provides forty pairs of attachment and pulling states (*n_f_*(*t*), *n_b_*(*t*)) and (*q_f_*(*t*), *q_b_*(*t*)). Using such data it is possible to build velocity histograms corresponding to the different motor states. In this way, we are able to discriminate the contribution of these motor states to the total velocity distribution. Figure S1 sketches this analysis for pulling states.

## Results

We analyzed a large number of simulated trajectories for many different sets of parameters values. This allowed us to have an important insight of the behavior of the model when varying the different parameters, and also to find sets of parameters compatible with the results for melanosomes transport. We must remark that, since the model is highly non- linear and the parameter space dimension is large, a systematic exploration is impracticable.

Thus, in this paper we consider three different approaches. In the first one, we analyze symmetric configurations with *N_f_ = N_b_* in which both motor types have the same single-motor parameters but differ in their stepping direction. These considerations reduce significantly the parameter space and facilitate the analysis of many dynamical properties which are expected to change smoothly when abandoning symmetry. Within this symmetric approach we obtain velocity distributions and cargo trajectories similar to those reported for melanosomes [1], [8], [38].

Then, we consider close-to-symmetric configurations with unequal attachment and detachment rates for forward and backward motors, but keeping the rest of the parameters symmetric. We will use this approach to analyze aggregation and dispersion processes in melanophores, where the dominance of one motor species is essential. Note that close-to-symmetric conditions –i.e. comparable number of kinesins and dyneins with similar stall forces- have been reported for *in vivo* systems [18], [35], [36].

Finally, in order to take into account the *in vitro* results [30], [31], [32] indicating that dynein stall forces are much lower than those of kinesin, we analyze asymmetric situations with *N_f_* = 1, *N_b_* = 5 and *F_sf_*∼5 *F_sb_*. This asymmetric situation has also been proposed for endosomes transport by dynein and kinesin-1 *in vivo* [10]. We will show that this consideration leads to velocity distributions with characteristics that strongly differ from those obtained for melanosomes, probably due to the fact that the transport is driven by different motors of the kinesin family.

In most of our paper we use the parameters *v_0_* = 500 nm/s and *F_s_* = 6 pN [16], [17], [22], [33], [34], [41], and we focus on analyzing the role of the remaining parameters. However, we show that our main results and conclusions remain essentially unchanged when considering lower values of the stall force in the range F_s_>2.5 pN (as those reported for *in vivo* systems in [35], [36]), after a slight rescaling of some parameters (see Information S2).

### General properties of the velocity distributions

We constructed histograms of segmental velocities of the cargo as described before. Due to the symmetry assumption, the velocity histograms are equivalent for cargoes moving in both directions. So, we restrict our analysis mainly to pieces of trajectories that move toward the positive direction and computed the average velocities for these segments only.

**Figure 3:**
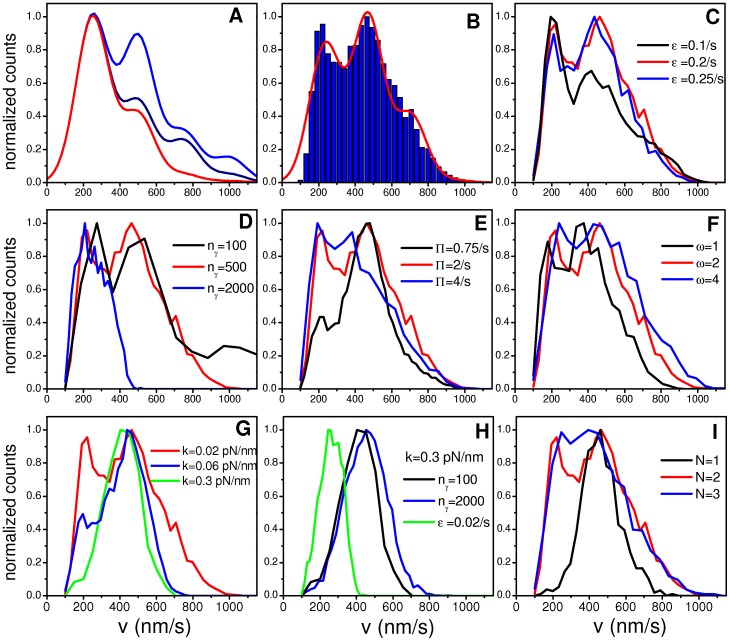
Distributions of cargo segmental velocities. (A) Experimental results from reference [8] corresponding to motion towards MT minus-end during aggregation (light blue) and dispersion (dark-blue), and motion toward the MT plus-end during either aggregation or dispersion (red). The three curves are fits of the experimental histograms by four Gaussian functions (Eq. S1). (B) Distribution of segmental velocities obtained from simulations using the symmetric referential parameter set (RS) indicated in Table 1. The bars represent the histogram while the red curve corresponds to the fitting with Eq. S1. (C)–(I) Histograms from simulations for symmetric teams with all the parameters as in the RS excepting for those specifically indicated in the panels. In all the cases from (C) to (I), the red curve is for the RS. The normalization used for all the distributions is the same as in [8], with the maximum of the distribution set equal to 1.

To make the comparison between experimental and simulated data easier, we included in Figure 3A the experimental velocity distributions determined for melanosome transport by microtubule motors in *Xenopus laevis* melonocytes [8]. To facilitate the visual observation of the data, Figure 3A shows the distributions both for plus-end and minus-end directed motion in the positive axis.

Figures 3B–I show the distributions obtained from simulations with several different sets of parameters. In this section, we discuss some general aspects concerning the emergence of multimodal distributions, while the detailed analysis of the role of each parameter will be presented later, when studying the influence of the pulling and attachment states on the velocity distributions.

The parameter set used in Figure 3B and specified in Table 1 will be considered as a *referential set* (RS). It should be stressed, however, that this does not correspond to a special fit of the experiments, but it is just a representative set that results in a distribution with characteristics similar to the experimental ones. As it can be seen, the distribution is multimodal with local maxima centered at multiples of ∼250 nm/s, which is in agreement with the experimental results.

**Table 1:** Reference parameter set (RS) for symmetric motors.

**Environment and cargo parameters**		
number of motors	N_f_ = N_b_	2
viscosity/water viscosity	n_γ_	500
organelle radius	r	500 nm
temperature	T	300 °K
**Single motor parameters**		
minimal distance for pulling	x_0_	110 nm
motor stiffness	k	0.02 pN/nm
zero-load velocity	v_0_	500 nm/s
exponent of f-v relation	w	2
stall force	F_s_	6 pN
zero-load detachment rate	ε	0.2/s
detachment force	F_d_	3 pN
attachment rate	Π	2/s

Figures 3C–F show the effects of varying the parameters ε, Π, n_γ_ and *w* with respect to RS, respectively. As it can be seen, the distributions remain multimodal in most of the studied conditions. However, very large variations of some parameters values (such as considering n_γ_ = 2000) can lead to the disappearance of the multimodal structure (green histogram in Fig. 3E). Multimodality is also conserved when parameters *v_0_* and *F_d_* are changed within certain reasonably ranges (results not shown). The same occurs for variations of *F_s_* in the range 1–6 pN, as we will show below in the corresponding section.

Interestingly, one of the most relevant parameters in determining the multimodal characteristic of the velocity distributions is the motor linker stiffness. Figure 3G shows that the distribution becomes unimodal when the parameter *k* is increased, due to a gradual decrease of the low velocity mode. The effect will be explained in detail in the next section. Importantly, we see that the model predicts a unimodal distribution for a stiffness value of 0.3 pN/nm, which is that determined for motors in *in vitro* assays [27], [28], [29] and used in most of the previous stochastic models [17], [23]. Figure 3H shows that this is also the case if we consider *k* = 0.3 pN/nm and other values for the parameters n_γ_ and ε.

Our results for several additional parameters sets (not shown) indicate that stiffness values *k*>0.1 pN/nm produce always unimodal distributions, while values of *k* in the range 0.01 pN/nm to 0.06 pN/nm lead to multimodal distributions under rather general conditions. These small stiffness values for individual motors are compatible with the results obtained in [1], where the authors have found that the stiffness for the whole melanosome-MT linking complex (involving possibly many motors) is distributed in the range 0.01 pN/nm–0.1 pN/nm.

Finally, Figure 3I shows the effect of varying the number of motors on the velocity distribution. The multimodality is lost for *N_f_ = N_b_ = *1, while in the case *N_f_ = N_b_ = *3, the distribution of velocities shows a single broad peak. The origin of this blurring effect will become clearer in the following sections when we analyze how the pulling states determine the cargo velocity.

A remarkable observation is that most distributions, especially those with small *k*, show relevant contributions at velocities much larger than *v_0_*, which represents the maximum velocity of advance for unloaded single motors. This result, which seems essential to explain the experimental distributions at high velocity values, will be further analyzed in the following sections.

### Analysis of the motor states contribution to the macroscopic behavior of the cargo

In the previous section, we showed how different parameters considered in the model affect the cargo velocity distributions. To better understand this behavior we analyze the correlation between the segmental cargo velocity and the motors states.

**Figure 4:**
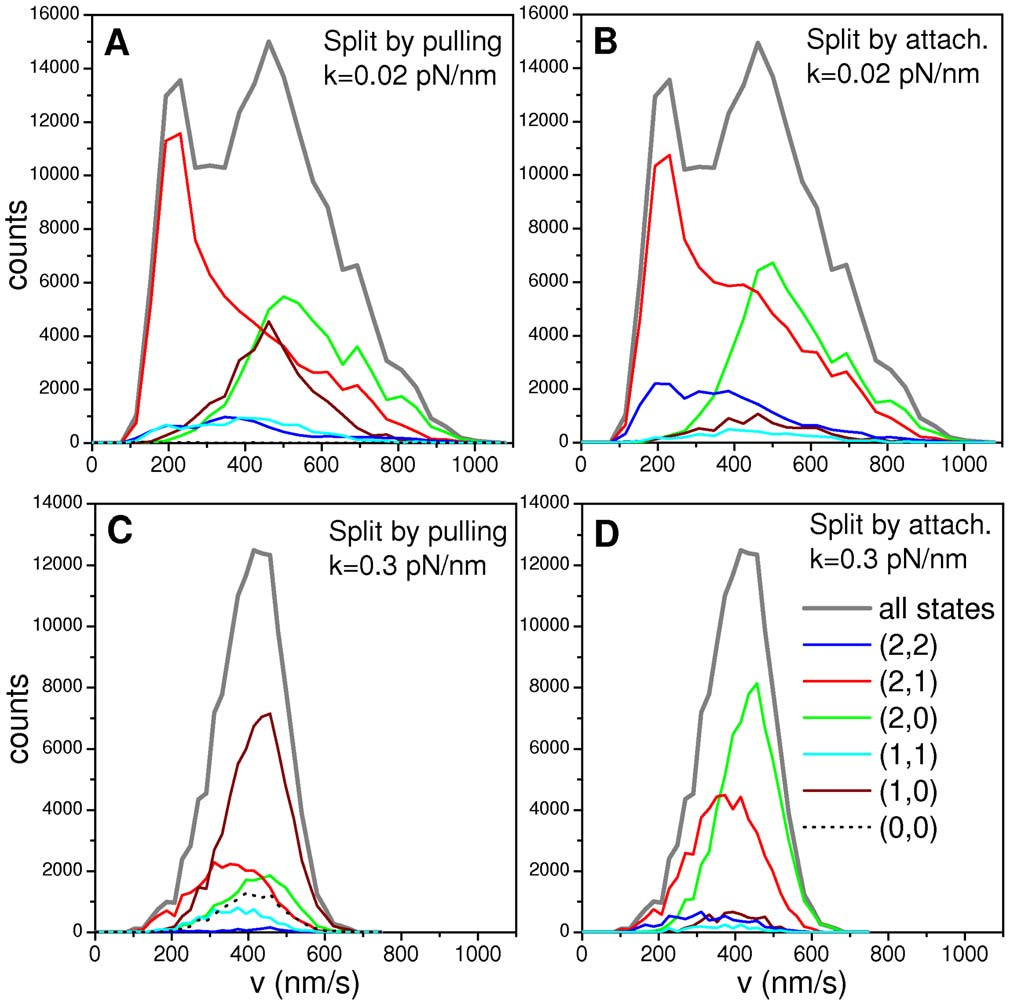
Pulling and attachment states contribution to the velocity distribution. (A) Contribution of the pulling states to the velocity distribution for the RS studied in Figure 3B (with *N_f_ = N_b_ = *2 and *k* = 0.02 pn/nm). (B) Ibid panel (A) for the attachment states. (C) Contribution of the pulling states to the velocity distribution for the system with *N_f_ = N_b_ = *2 and *k* = 0.3 pn/nm shown in Figure 3G. (D) Ibid panel (C) for attachment states. For all the panels, the indications are those in panel (D). The colored curves indicate the contributions of the different states to the overall distribution (gray curves), that are labeled as “all states”.

Let us consider the velocity distribution obtained for the RS and shown in Figure 3B. Figure 4A and 4B display the contribution of the different pulling and attachment states to the velocity histogram, respectively.

Figure 4A shows that the low velocity peak is mainly due to the contribution of the pulling state (*q_f_*, *q_b_*) = (2,1). In contrast, the peak at v∼500 nm/s is mainly due to the pulling state (*q_f_*, *q_b_*) = (2,0) but has also contributions from the pulling states (*q_f_*, *q_b_*) = (2,1) and (*q_f_*, *q_b_*) = (1,0). In addition, we see that the pulling state (*q_f_*, *q_b_*) = (2,0) contributes to the distribution at high velocities (>800 nm/s). Finally, it can be seen that pulling states (*q_f_*, *q_b_*) = (2,2) and (*q_f_*, *q_b_*) = (1,1) produce very small contributions spread in a relatively wide range of velocities.

Figure 4B shows that the attachment states (*n_f_*, *n_b_*) = (2,1) and (*n_f_*, *n_b_*) = (2,2) contributes to the low velocity peak. Taking into account the results shown in Figure 4A, this means that the attachment state (*n_f_*, *n_b_*) = (2,2) acts mainly through the pulling state (*q_f_*, *q_b_*) = (2,1), i.e. with one unloaded backward motor which must be thus localized at a distance from cargo smaller than *x_0_*.

By comparing Figure 4A and 4B, it can also be noticed that the contribution of the pulling state (*q_f_*, *q_b_*) = (1,0) to the cargo velocity histogram results from the motor attachment states (*n_f_*, *n_b_*) = (2,0) and (2,1), while the attachment state (*n_f_*, *n_b_*) = (1,0) is rather infrequent.

In the previous section, we have observed that the model predicts a unimodal distribution for the stiffness value of ∼0.3 pN/nm. In order to understand this, we analyze the contribution of the different motor states to the velocity histogram for *k* = 0.3 pN/nm previously shown in Figure 3G. As it can be seen in Figure 4C, the contribution of the pulling state (*q_f_*, *q_b_*) = (1,0) is dominant. In contrast, the pulling state (*q_f_*, *q_b_*) = (2,1), which was the responsible for the low velocity peak for low stiffness, results infrequent with a stiff motor linker. This stems on the fact that, for high stiffness, active tug-of-war leads to large forces which produce the rapid detachment of motors and, thus, biases the system toward single motors configurations.

Interestingly, the single peak observed in Figure 4C (high *k* value) corresponds approximately to the second peak of Figure 4A since both are centered at v∼500 nm/s and have similar widths. However, for low *k* the pulling state (*q_f_*, *q_b_*) = (2,0) dominates the ∼500 nm/s peak, while the pulling state (*q_f_*, *q_b_*) = (1,0) prevails for high *k*.

The origin of such behavior becomes quite evident when comparing Figures 4C–D; the attachment state (*n_f_*, *n_b_*) = (2,0) results in the pulling state (*q_f_*, *q_b_*) = (1,0) due to the fact that large stiffness prevents motors to get to distances much larger than *x_0_* from the cargo.

In short, our simulations show that low motor linker stiffness (typically *k*<0.06 pN/nm) allow the presence of tug of war states, such as the pulling state (*q_f_*, *q_b_*) = (2,1), that contribute to the low velocity peak in the velocity distribution. In contrast, for stiffness values >0.1 pN/nm, tug of war states are infrequent due to rapid detachment processes and, thus, we always found single modal distributions as those shown in Figure 3G–H centered at the zero-load velocity of single motors (*v_0_* = 500 nm/s).

At this point, it is easy to understand the dependence of the velocity distribution on ε and Π, analyzed in Figure 3C–D. The amplitude of the low-velocity peak increases when decreasing the detachment rate or increasing the attachment, since such changes in the parameters favor the occurrence of the pulling state (*q_f_*, *q_b_*) = (2,1).

In the previous section we also noted that the distribution of cargo velocity is unimodal when *N_f_* = *N_b_* = 3 (Figure 3I). However, this is not due to the absence of the low-velocity mode (as happened when increasing *k*), but to the blurring or collapse of the different modes in a single, wider peak. This blurring effect can be understood in terms of the contribution of the pulling states to the velocity histogram (Figure S2). We found that the tug of war pulling states (*q_f_*, *q_b_*) = (2,1), (3,1) and (3,2) are mainly responsible for low velocities while pulling states (*q_f_*, *q_b_*) = (2,0) and (3,0) are relevant only for large velocities. However, due to the large number of states, the two velocity modes merge into a single one.

Finally, Figure S3 shows the analysis of the data obtained for *N_f_ = N_b_ = *1. As expected, the single velocity peak is mainly due to the pulling state (*q_f_*, *q_b_*) = (1,0).

### Spatial distribution of motors and force production

Since the cargo velocity is directly linked to the net force exerted by the motors (Eq. 2), and the motor-cargo interactions are distance dependent (Eq. 1), the average motion of the cargo is related to the motors positions along the MT. Therefore, we study the spreading of the motors around the cargo position for different cargo velocities and different stiffness values of the motor linker. Clearly, this gives information about the force produced by the pulling motors during the transport.

To determine the spatial distributions of motors around the cargo position for forward and backward motors, we proceed as follows. For a given parameter set, we selected intervals of the cargo velocity and computed the positions of the motors at every point of each 40-data trajectory segment included in the considered velocity interval.

Since the motor stiffness plays an essential role in regulating the strength of the force exerted by the motors, we focus our analysis on the two systems analyzed in Figure 4. Namely, the RS (with *k* = 0.02 pN/nm) and the system with *k* = 0.3 pN/nm, also studied in Figure 3G.

**Figure 5:**
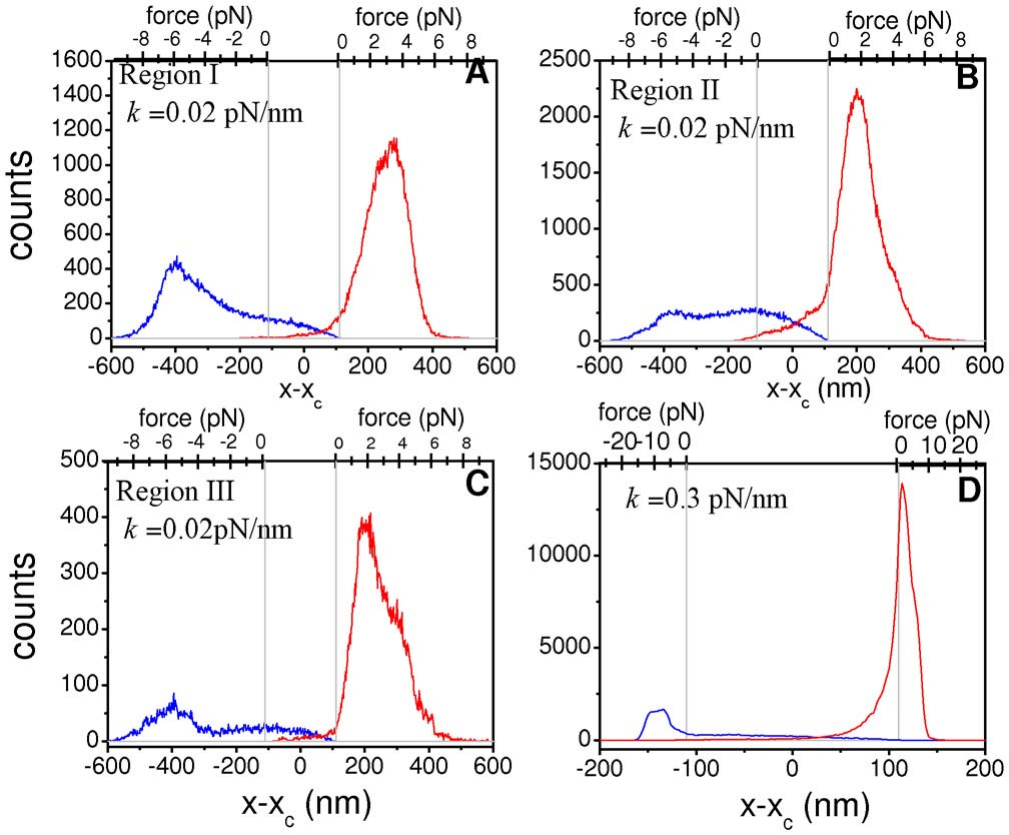
Spatial distributions of motors around cargo position for different intervals of the cargo velocity. In all panels, the red and blue curves indicate the distribution of forward and backward motors, respectively. The force scales at the top axis indicate the force exerted by a motor located at the corresponding position. The vertical gray lines indicate the limit positions *x−x_c_* = ±110 nm separating regions of pulling and non-pulling motors. Panels (A), (B) and (C) show the results for the RS (*k* = 0.02 pN/nm) considering the velocity regions I, II and III defined in the main text, respectively. Panel (D) shows the results for the system with *k* = 0.3 pN/nm studied in Figure 3G considering the region 300 nm/s<*v*<500 nm/s. Note that, in all the cases, the data were taken from segments with positive (forward) velocity, when the cargo is moving to the right. The parameter μ, defined in the main text, is computed as the quotient between the area below the red histogram for *x−x_c_*>110 nm and the area below the blue one for *x−x_c_*<−110 nm.

The multimodal velocity distribution obtained for RS suggests the consideration of three natural velocity intervals. We define region I for 100 nm/s<v<300 nm/s, corresponding to the low velocity peak, region II for 350 nm/s<v<550 nm/s covering the second peak, and region III as 700 nm/s<v<900 nm/s capturing the large velocity tail. Thus, Figure 5A, 5B and 5C show the spatial distributions of motors around the cargo for regions I, II and III, respectively. On the other hand, for *k* = 0.3 pN/nm we consider only one region centered at the single mode of the velocity distribution, delimitated by 300 nm/s<v<500 nm/s. The corresponding distribution of motors is plotted in Figure 5D. The force scales for forward and backward motors are indicated at the top of each panel. Note that for positions in the range −110 nm<*x−x_c_*<110 nm the motors do not exert forces on the cargo.

Interesting results can be extracted from Figure 5. First, the spatial distribution of motors narrows considerably for high values of *k*. This is a consequence of the larger forces supported by the motors at a fixed distance from cargo. In addition, it can be observed that for *k* = 0.3 pN/nm nearly half of the attached forward motors are not pulling motors. In contrast, for *k* = 0.02 pN/nm, most attached motors are also pulling motors.

Figures 5A–C also indicate that the proportion of pulling backward motors is much larger at low cargo velocities. To quantify this effect we define the parameter μ, which represents the ratio between the number of pulling forward motors and that of pulling backward ones (see definition of μ in Figure 5 caption).

We obtain μ values of 1.9, 3.8 and 4.7 for the distributions shown in Figure 5A–C, respectively. The particularly low value of μ found for region I shows that the cargo velocity decreases when the two teams of motors are involved in tug-of-war since this process leads to a low *net* force exerted on the cargo. Furthermore, the individual forces exerted by the motors are considerably stronger for region I than for the other cases. For instance, note that the forces exerted by forward motors are approximately equal to 3.5 pN (Figure 5A) while for the other cases the forces are ∼2 pN (Figure 5B–C).

Note that the value μ = 1.9 obtained for region I is compatible with the dominance of the pulling state (2,1), for which we would expect μ = 2. In contrast, cargoes in regions II and III owe their faster motion to the lower proportion of pulling backward motors. On the other hand, we obtain μ = 4.8 for the system with *k* = 0.3 pN/nm indicating that the presence of pulling backward motors is not frequent at high stiffness.

Figure 5 also shows that the backward motors are located further from the cargo and thus support larger forces than forward motors. According to our model, backward motors are unable to move when they surpass the critical distance *x_0_*+*F_s_*/*k* at which their load force equals *F_s_*. If the cargo continues moving forward, the distance between the cargo and backward motors increases until a detachment process occurs leading to a change in the configuration of the system and thus in the overall dynamics. Thus, our model predicts that the backward motors act effectively as elastics anchors for the cargo motion as was previously suggested [23], [42].

### Results for lower values of the stall forces

In the above sections we have considered *F_s_* = 6 pN for both motor species. In this section we analyze the results of the model simulations with lower values of the stall force. The rest of the parameters were slightly changed according to the scaling procedure described in Information S2.

**Figure 6:**
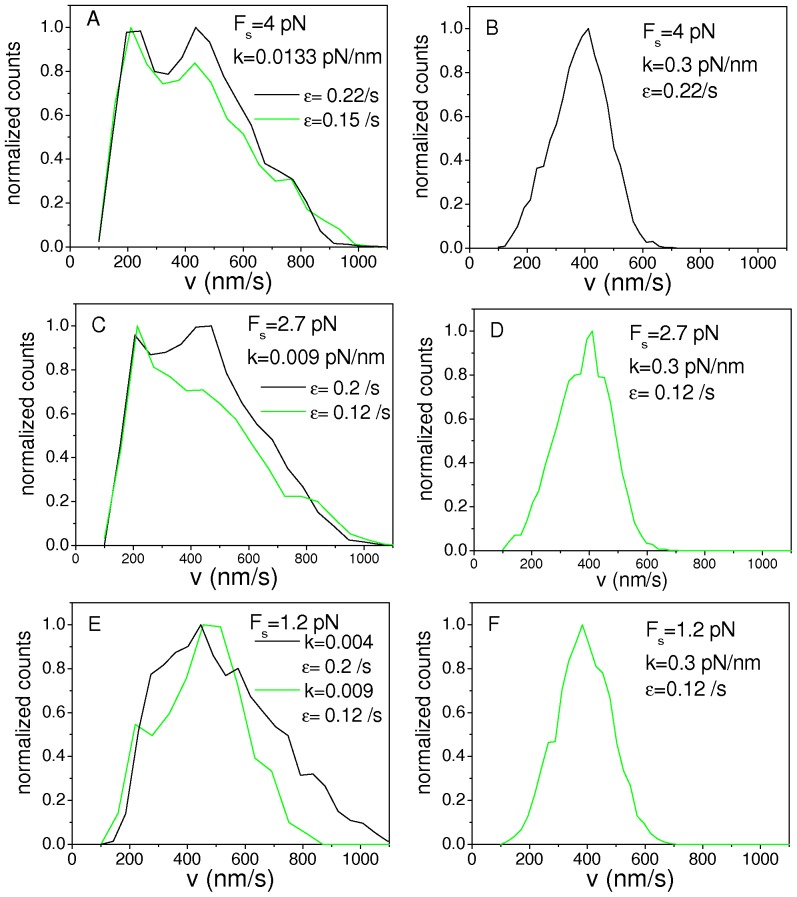
Velocity distributions for symmetric systems at lower stall forces. Velocity distributions for symmetric systems with: (A)–(B) *F_s_* = 4 pN, n_γ_ = 333 and *F_d_* = 2.4 pN (i.e. transformed from the RS using eq. S2 with λ = 2/3 and β = 0.8), (C)–(D) *F_s_* = 2.7 pN, n_γ_ = 225 and *F_d_* = 1.7 pN (i.e. transformed from the RS using eq. S2 with λ = 0.45 and β = 0.57) and (E)–(F) *F_s_* = 1.2 pN, n_γ_ = 100 and *F_d_* = 1.34 pN (i.e. transformed from the RS using eq. #2 with λ = 0.2 and β = 0.447). In all the cases, the values of *k* and ε are indicated in the panels while the remaining parameters are those in Table 1.

Figure 6 shows the segmental velocity distributions for symmetric systems with *N_f_ = N_b_ = *2 considering different values of *F_s_*, ε and *k*. Importantly, we found that for *F_s_* in the range 1.2–4 pN the velocity distributions change from being multimodal (Figure 6A, 6C and 6E) to unimodal (Figure 6B, 6D and 6F) when the individual motor linker stiffness is increased from ∼0.01 pN/nm to ∼0.3 pN/nm, as was observed for *F_s_* = 6 pN. This result suggests that the motor linker stiffness is a key parameter in determining the shape of the distributions, as mentioned in the previous sections.

Furthermore, the distributions obtained for *F_s_* = 4 pN and *F_s_* = 2.7 pN (Figure 6A and 6C), as well as their dependence with the detachment rate are very similar to those obtained for *F_s_* = 6 pN (Figure 3), and thus the main results discussed in the previous sections remain essentially unchanged when considering values of the stall forces from ∼2.5 pN to 6 pN.

On the other hand, we were not able to reproduce the shape of the velocity distribution obtained for melanosomes with *F_s_* = 1.2 pN (Figure 6E–F). However, we cannot ensure that this would the case for other parameters sets not considered here.

### Reversions of the direction of motion

In the previous sections we have shown that our model is able to generate velocity distributions compatible with those observed for transport of melanosomes in *Xenopus* melanophores [8]. In this section, we show that the proposed model is also able to reproduce the characteristic wave-shape reversion patterns observed during melanosome transport along microtubules [38].

With this aim, we analyzed in detail selected regions of the simulated trajectories where the cargo travels back for at least 250 nm during more than 1 s (i.e. long-term reversions [38]).

**Figure 7:**
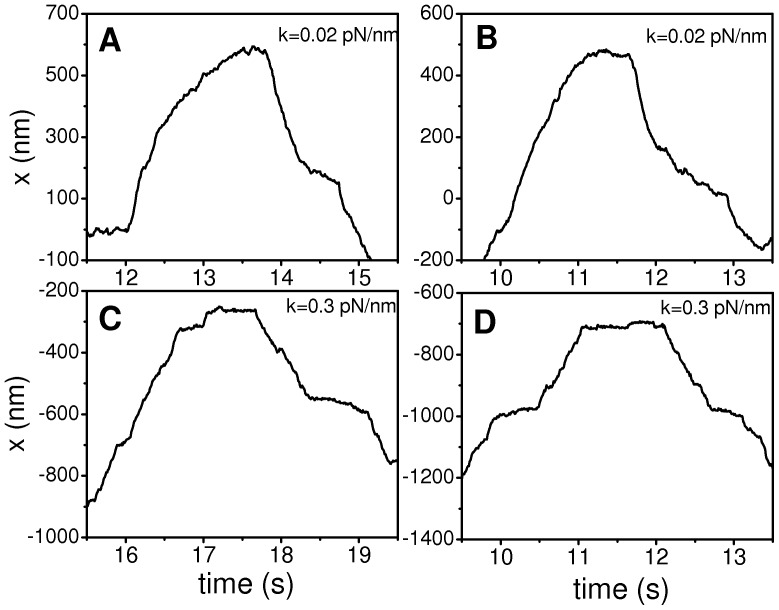
Representative reversions for systems with large and low motor stiffness. Trajectories in panels (A) and (B) correspond to the RS (stiffness *k* = 0.02 pN/nm), while those in panels (C) and (D) correspond to the system with *k* = 0.3 pN/nm studied in Figure 3G.

Figure 7 shows representative time courses of reversions taken from simulated trajectories considering either soft (0.02 pN/nm) or stiff (0.3 pN/nm) motor linkers. In the first case, reversions are similar to the experimental wave-shaped reversions described in reference [38]. On the contrary, stiff motor linkers lead to abrupt reversions in which the cargo suddenly stops before switching the direction of motion.

Our studies indicate that wave-shape reversions could not be reproduced when considering motor-linkers stiffness higher that 0.1 pN/nm even if other parameters values of the model were modified (e.g. see Figure S8). Moreover, wave-shaped reversions are a common observation at low *k* for different parameters sets, including asymmetric cases (*N_b_≠N_f_*, *v_0b_≠v_0f_*, *ε_b_≠ε_f_*, etc.).

In order to quantify the difference between reversions obtained for different stiffness, we focused on the characteristic time of the slowing down process. For each analyzed reversion, we fitted the time course of the position of the organelle before the reversion point with the following expression [38] (see Fig. S4):
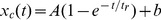
(5)where *A* and *b* are constants and *t_r_* is the rising time of the reversion.

The recovered distributions of *t_r_* followed exponential decays (not shown), with characteristic values of 280±90 ms for the RS. This value is not significantly different from that determined during melanosome transport (∼300 ms, [38]). On the contrary, *t_r_* was 95±30 ms for sets with *k* = 0.3 pN/nm.

**Figure 8:**
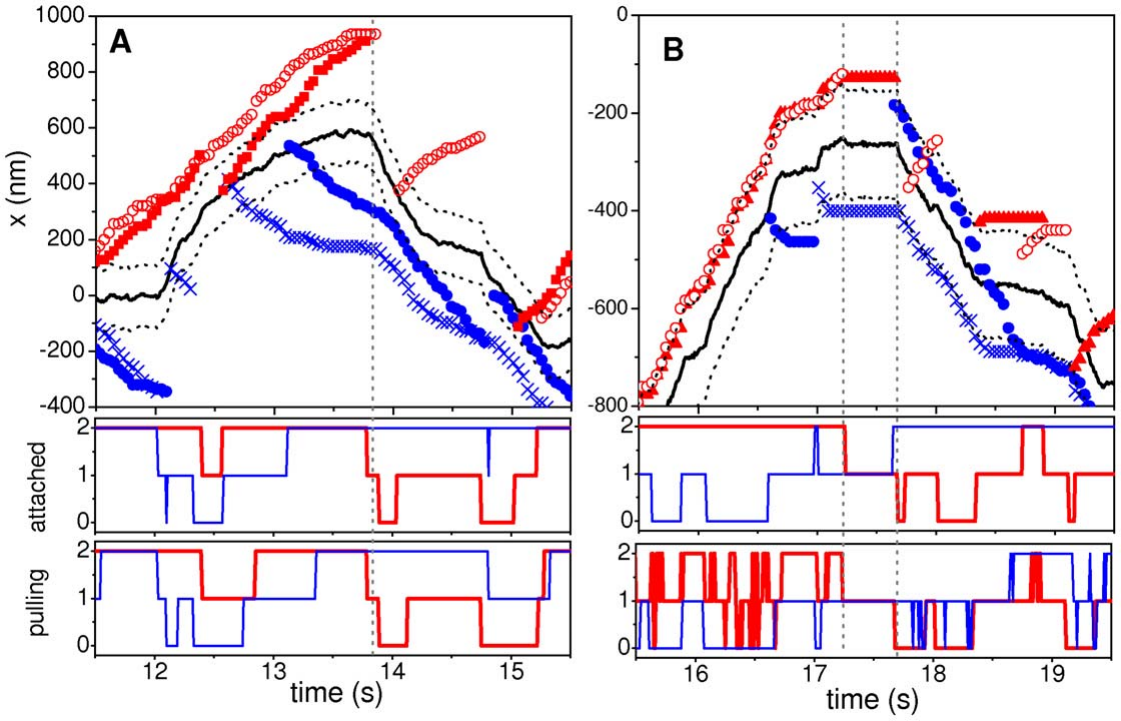
Trajectories of cargo and motors during reversions. (A) Details of the reversion shown in Figure 7A (RS, *k* = 0.02 pN/nm). (B) Ibid for the reversion shown in Figure 7C (system with *k* = 0.3 pN/nm). In both panels, the cargo trajectories are indicated by thick solid lines while different red and blue symbols are used for forward and backward motors, respectively. The limits for pulling (positions *x_c_*(*t*)±*x_0_*) are indicated by dotted lines. The bottom panels indicate the numbers of attached and pulling motors of both species as function of time during the reversion. The vertical dotted segments indicate relevant detachment events for the reversions.

To better understand the dynamical processes underlying reversions, we analyzed the positions of the attached motors during these events (Figure 8), as well as the time dependence of the number of attached and pulling motors (see also Figures S5 and S6).

Interestingly, for small *k* (Figs. 8A and S5), the long-lasting slowing down process observed before the reversion is due to a long-lasting unbalanced tug-of-war that becomes gradually balanced. The reversion is then triggered by the detachment of a forward motor which leads to a fast backward motion of the cargo due to the elastic restitution of the motor-linker after the detachment. The cargo then resumes its motion with a slower velocity.

On the other hand, for larger values of *k* (Figures 8B and S6), motors are not allowed to explore large regions beyond the thresholds *x_c_*±*x_0_* and, consequently, the restitution processes have short durations. This leads to the more abrupt changes observed on the cargo velocity, which are essentially controlled by attachment and detachment events. It is also interesting to note that, for large *k*, the number of pulling motors fluctuates much more rapidly than that of attached ones, since motors goes back and forth the limits *x_c_*±*x_0_*. In contrast, for small *k*, the numbers of attached and pulling motors are highly correlated.

The analysis of trajectories and detachment events also sheds light on the origin of the large tails of the velocity distributions obtained for small *k* (see Fig. 3). In fact, we found that the segments with velocities much larger than *v_0_* correspond to elastic restitutions induced by detachment after strong tug-of-war processes. The effect is illustrated in Figure S7, where we show several high velocity segments triggered by detachment events.

On the other hand, for large values of *k*, although the instantaneous velocity after detachment is also large, elastic restitutions are fast. Thus, these segments do not satisfy the selection criteria to be included in the segmental velocity distributions. So, velocities much larger than *v_0_* do not contribute to the segmental velocities histograms.

### Dispersion and aggregation in Xenopus laevis melanophores

*Xenopus* melanosomes disperse in the cytoplasm or aggregate in the perinuclear region when stimulated with specific hormones [43], [44]. In cells treated with Latrunculin B (a drug that depolymerize actin filaments), the movement is entirely microtubule-dependent, and melanosomes are carried by plus-end-directed microtubule motor kinesin-2 [45] and the minus-end-directed motor cytoplasmic dynein [46]. It has been demonstrated that certain hormones such as melatonin and MSH trigger the motion of these organelles toward the cell nucleus or the cell periphery, respectively [44], [47].

It has been found that the organelle segmental velocities distributions were multimodal with maxima located at the same velocity values in both stimulation conditions [8]. Similar distributions of segmental velocities were observed for organelles moving toward the plus end of the microtubule. On the contrary, the organelles moving toward the minus end increased the probability of developing large velocities when treated with melatonin (Figure 3A).

In order to account for these observations, we generated trajectories with our model modifying some of the parameters values with respect to the RS. In particular, it was necessary to abandon the symmetry condition, since the experimental distributions for forward and backward segmental velocities are different. The fact that the positions of the maxima of the experimental velocity distributions are the same for the plus and minus-directed organelles [8] suggests that *v_0_* and *F_s_* should be similar for the two motor species. Therefore, we focused our analyses on studying the modifications on the velocity histograms introduced by changing the attachment and detachment rates (Π and ε, respectively) of one of the motor families or altering the ratio between *N_f_* and *N_b_*.

Our results showed that small imbalances of the number of motors introduced a dramatic bias of cargo motion in a defined direction. For example, when considering *N_f_* = 2 and *N_b_* = 3, we observed 98% of backward-directed segments. Similar results were obtained for *N_f_* = 1 and *N_b_* = 2. In contrast, the percentage of minus-directed melanosome motion observed in the experiments is about 60 and 40% during aggregation and dispersion, respectively [8]. Thus, we conclude that an imbalance in the number of motors cannot explain the different velocity distributions of dynein-driven melanosomes during aggregation and dispersion.

**Figure 9:**
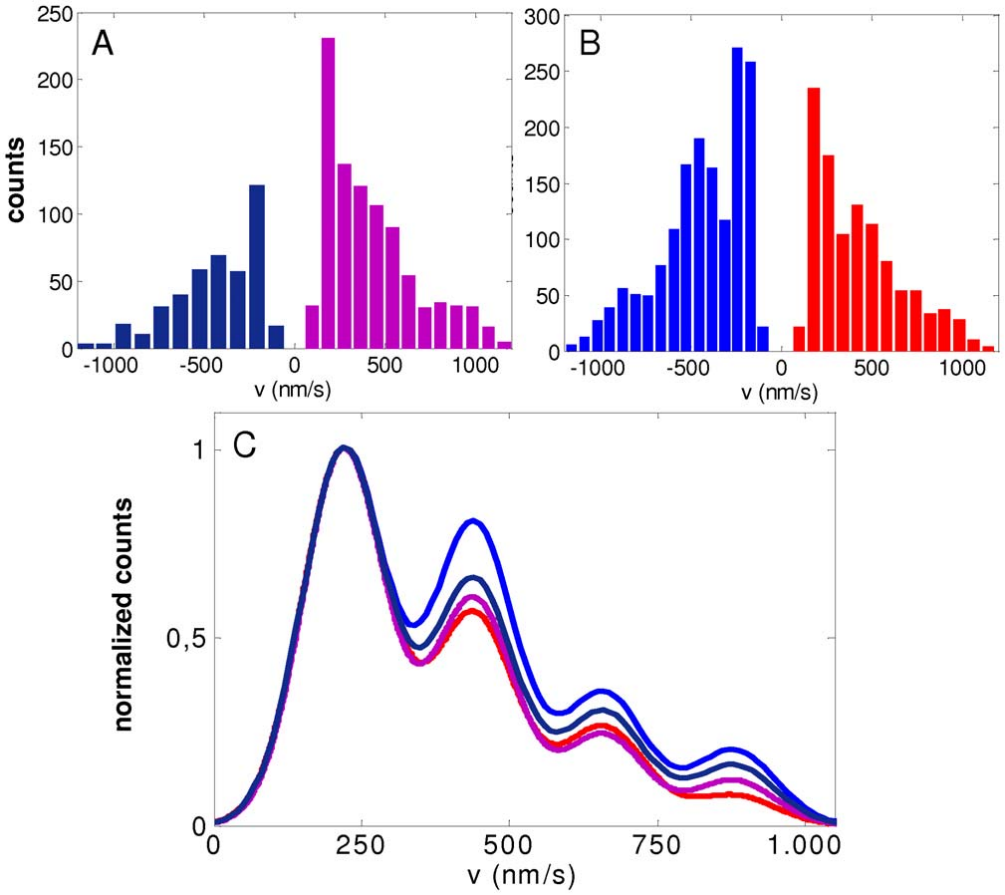
Modeling aggregation and dispersion regimes. Histograms of segmental velocities obtained from simulations with non-symmetric motor teams considering: (A) Parameter Set 1: π_f_ = 3.5/s, π_b_ = 2.2/s, ε_f_ = 0.08/s and ε_b_ = 0.07/s. (B) Parameter Set 2: π_f_ = 1.8/s, π_b_ = 2.2/s, ε_f_ = ε_b_ = 0.08/s. The remaining parameters of Set 1 and Set 2 are those of the RS (Table 1), excepting for the value of the viscosity for which now we consider n_γ_ = 300. Panel (C) shows the fits by Eq. S1 of the positive and negative velocity branches of the histograms in panels (A) and (B), computed as explained in Information S3. The magenta and the dark-blue curves are the fits of the forward and backward branches of the distribution in panel (A), respectively. The red and the light-blue curve are the fits of the forward and backward branches of the distribution in panel (B), respectively. The parameters of the fits in panel (C) are shown in Information S3.

Therefore, we independently changed the values of Π_f_, Π_b_, ε_f_ and ε_b_ with respect to those values included in the RS and determined the cargo velocities distribution in each condition. After several simulations, we selected the two parameter sets here referred to as Set 1 and Set 2 (see Table 2) whose corresponding velocity distributions are shown in Figure 9 (Fig. 9A–B). Importantly, Set 1 considers Π_f_>Π_b_ which favors forward motors, while Set 2 considers Π_b_>Π_f_ favoring backward motors.

**Table 2:** Dispersion and aggregation.

Set	% of backward directed segments	A_2_/A_1_ for backward directed segments	A_2_/A_1_ for forward directed segments
Experimental: dispersion [8]	32%	0.48±0.1	0.41±0.1
Simulated. Set 1	32%	0.61±0.18	0.55±0.15
Experimental: aggregation [8]	64%	0.87±0.18	0.41±0.1
Simulated. Set 2	60%	0.8±0.2	0.57±0.15

Summary of the experimental results obtained by Levi *et al.* [8] for melanosomes stimulated with MSH for dispersion and with melatonin for aggregation, and results for simulated trajectories given by Set 1 and Set 2 (corresponding parameters given in caption for Figure 8). A_2_/A_1_ is the ratio between the first and the second peak amplitudes obtained when fitting Eq. S1 to the histograms shown in Fig. 8A and 8B.

We normalized the histograms shown in Figure 9A–B, and fitted Eq. S1 (see Information S3) to these data (Figure 9C). Table 2 shows that the percentages of backward-directed segments obtained for the simulated trajectories agreed well with the experimental observations. Also the ratio between the amplitudes of the first and second mode (A_2_/A_1_) reproduces within the error the different A_2_/A_1_ ratios obtained for backward-directed segments when changing from dispersion to aggregation, as well as the invariance of A_2_/A_1_ for forward-directed motion.

Despite the slightly different value of ε_b_ (which improves the agreement with the experimental data), the main difference between the data sets considered in Figures 9A–B is the value of Π_f_ suggesting that changes in the attachment rate of kinesin motor may control the transition between dispersion and aggregation regimes.

The results shown in this section indicate that the attachment and detachment rates play a very crucial role in regulating the net direction of transport of the cargoes, as well as the profiles of the segmental velocities distributions. Moreover, they suggest that the stimulation conditions considered in [8] may affect the attachment and detachment probabilities.

### Simulations with two teams of motors with very different stall forces

Recently, Soppina *et al.* [10] have reported an asymmetric motor competition in *Dictyostelium* cells for which these authors estimate that a number of dyneins between 4 to 8 with F_s_∼1.1 pN work against 1 or 2 kinesins with F_s_∼5 pN. Although kinesin-1 motors drive endosomes in *Dictyostelium* cells, while melanosomes are carried by kinesin-2 motors, we wonder if this kind of asymmetric configuration of motors can give rise to the velocity distributions in melanophores cells.

**Figure 10:**
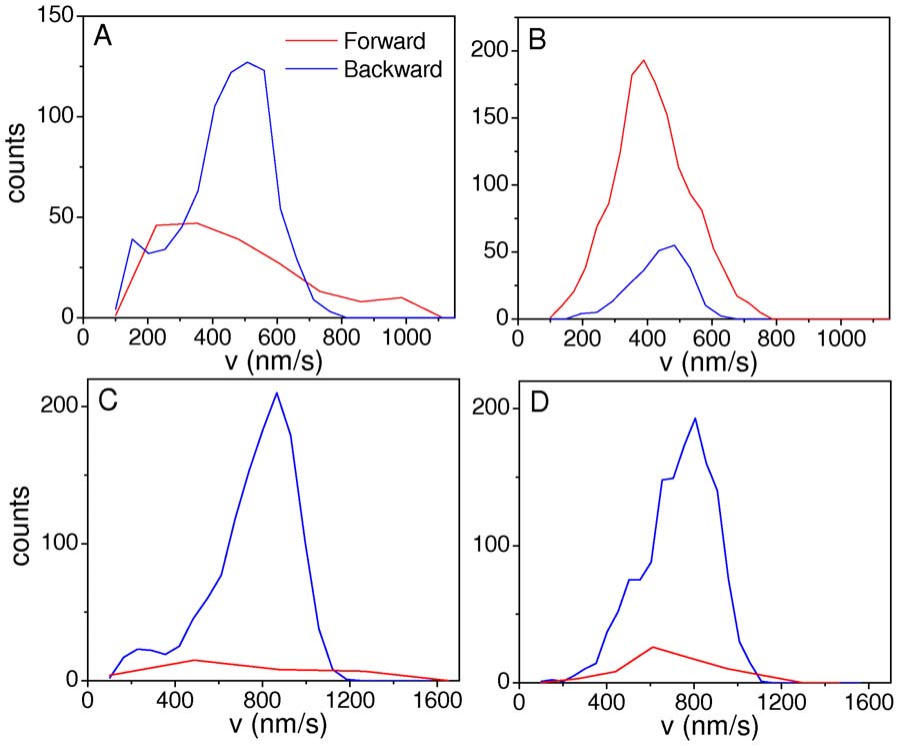
Velocity distributions for systems with strongly asymmetric stall forces. Segmental velocity distributions from simulations considering *F_sf_∼5 F_sb_* with *N_f_* = 1 and *N_b_* = 5. The red and blue curves represent the distribution for forward and backward velocity segments, respectively. A and C correspond to low motor linker stiffness (*k_f_ = k_b_* = 0.02 pN/nm) while B and D consider *k_f_ = k_b_* = 0.1 pN/nm. The remaining parameters are the following: A–B: *F_sf_* = 5 pN, *F_sb_* = 1.1 pN, *F_df_* = 3 pN, *F_db_* = 1 pN, *Π_f_* = 5/s, *Π_b_* = 1.6/s, *n_γ_* = 100. C–D: *F_sf_* = 5.5 pN, *F_sb_* = 1.1 pN, *F_df_* = 3 pN, *F_db_* = 1.5 pN, *v_0f_* = *v_0b_* = 1000 nm/s, *Π_f_* = 5/s, *Π_b_* = 1.6/s, *n_γ_* = 100. The rest of parameters are displayed in Table 1.

To this end, we performed simulations considering 5 dynein motors with F_s_ = 1.1 pN and one kinesin with F_s_ = 5 pN. In Figure 10 we show four representative distributions obtained with different values of the rest of the parameters.

In almost every considered set of parameters, the distributions change from being multimodal (Figure 10A and 10C) to unimodal (Figure 10B and 10D) when increasing *k*, as observed for the symmetric configurations studied in the previous sections.

However, the velocity distributions for forward and backward motion are very different from those found in the experiments (Figure 3A). In particular, although we tried different combinations of the rest of the parameters values, the center positions of the velocity peaks for forward and backward motion do not coincide. For example, in the distribution shown in Figure 10C we increase the values of the backward motors detachment force and *v_o_* for both teams with respect to the one shown in Figure 10A, as an attempt to shift the peaks of the distribution and make it wider. However, we did not succeed in reproducing the experimental results.

This analysis suggests that a model considering similar stall forces and similar number of motors for both species results in distributions that are in better agreement with the segmental velocity distribution of melanosomes driven by microtubular motors in *Xenopus laevis* melanophores than a very asymmetric one.

## Discussion

In this paper we presented a stochastic model to investigate the transport of cargoes along microtubules. The model offers plausible explanations about how the typical features observed in trajectories of cargoes *in vivo* are determined by the motors stochastic dynamics considering a small number of motors with different polarities and standard force-velocity relations. Our studies focused on reproducing and interpreting previous experimental results for Xenopus melanosomes transport in living cells.

The model reproduce the main characteristics of the multimodal velocity distributions observed for melanosomes transport [8], as well as the typical wave-shaped reversion patterns reported for cargo trajectories [38].

Importantly, the mean field models [16], by their own hypotheses, can neither produce cargo velocities larger than the zero-load velocity of single motors observed in the distributions, nor reproduce the non-constant velocity profiles observed in the slowing down processes occurring before wave shaped reversions.

In our studies, the stiffness of the motor-linkers emerges as one of the most crucial parameters. We found that the experimental velocity distributions and the wave shaped reversions can only be reproduced considering stiffness of the order of 0.02 pN/nm. In contrast, stiffer linkers results in narrower unimodal velocity distributions and abrupt reversion patterns. The value 0.02 pN/nm for the stiffness of individual motors is in good agreement with the values distributed around 0.06 pN/nm reported in [1] for the stiffness of the whole cargo-microtubule linkage, that would involve several motors.

The present unified interpretation of the experiments based on the tug-of-war effect differs from other explanations given before for velocity distributions [8] and wave shaped reversions [38]. This deserves some discussion.

In reference [8] Levi *et al.* proposed a simple heuristic model for explaining transport of melanosomes. In short, they interpreted the first peak of the distributions shown in Figure 3A of the present paper as originated by one forward motor, the second peak as originated by two forward motors and so on. This was done in 2006 in a context in which tug of war models were not considered as quite suitable for giving good descriptions of the experiments [3]. Now, our present work shows that if low values of the motor-linker stiffness are assumed, a tug of war model can explain the distributions. Our approach reinterprets the first peak of the distribution as produced mainly by cargoes with two forward motors and one backward motor engaged in an active tug of war, the second peak as originated by states with forward motors only, and the large velocity peaks as due to elastic restitutions occurring after detachment of backward motors.

Concerning wave-shaped reversions, the model in reference [38] assumed that the stiffness of the motor-linker was that reported in *in vitro* assays. With such consideration it was not possible to explain the wave-shaped reversions as due to tug of war effects. Thus, reversions were attributed to collisions with external obstacles. After that, in reference [1], the stiffness of the motor-linker in living cells was found to be lower than those measured *in vitro*. Now, considering such small stiffness values for the motor-linkers, we see that a tug of war model can also explain the reversions. Importantly, note that both mechanisms for reversions (collisions and tug of war) could coexist, and thus this issue deserves further investigation.

Agreeing with our statement about the importance of considering motor linkers with low stiffness, Bieling *et al.* [20] observed in gliding assays of kinesin-1 motors that a rigid mechanical coupling can cause mutual interference when motors are mechanically coupled.

Recently, Soppina *et al.* [10] reported that endosomes inside *Dictyostelium* cells slow down and elongate during reversals due to the action of opposite motors applying force against each other. This raises the question whether melanosomes elongate or not during wave-shaped reversals observed in melanophore cells. Guo *et al.* [48] measured the Young modulus of melanosomes and found that they are considerably higher than the modulus of organelles with cytoplasm (∼1 MPa) and approaching values of the modulus of protein crystals (∼100 MPa). Despite these measurements were done on melanosomes of human retinal pigment epithelium, their properties are not expected to be very different from those of *Xenopus laevis* melanophores and thus the latter can be considered as stiff organelles. Moreover, we have to mention here that we did not detect deformations of melanosomes during reversions.

Other related studies by Ali *et al.* [42] recently studied the transport of single quantum-dot-labeled myoV and myoVI motors linked to a common cargo and showed that the velocity distribution of these complexes is multimodal, in contrast to the single-peak distribution observed for either myoV or myoVI alone. Furthermore, when varying the ratio of myoV∶myoVI, the heights of the peaks change accordingly. They interpreted these results according to a tug-of-war model. In the same direction, they found that the stepping rate of the winning motor slows down due to the resistive load of the other motor, which acts as an anchor. Clearly, these findings share strong similarities with our results.

Our model also shows that the detachment and attachment rates can regulate the net direction of transport as well as the relative weights of the different motors states. In this direction, we could reproduce the experimental velocity distributions obtained for melanosome transport during dispersion or aggregation [8] by only considering in the model a higher value for the attachment rate of forward motors in dispersion conditions. Relatively small variations in detachment and attachment rates resulted in important changes in the velocity histograms mainly associated with the prevalence (or not) of configurational states where the motors are involved in tug-of-war processes. In the present work, we assumed a standard exponential dependence of the detachment probabilities on the load. The consideration of more complex non-monotonous relations, as those proposed in recent theoretical [24] and experimental [18] works for related systems, could have seem rather artificial within our approach, since there is not detailed knowledge of the detachment rates for kinesin 2 and dynein in melanophores. However, our results highlight the importance of understanding the details of the detachment mechanisms.

As mentioned in the Introduction, there is some controversy in the literature regarding the values of the stall forces. In the case of dynein, different *in vitro* experiments report values in the range ∼1 pN [30], [31], [32], while other asserts values ∼7 pN [33], [34] (although the report in [34] corresponds to yeast dynein which may be different). Meanwhile, in living cells, values around 2.5 pN [35] and in the range 3–5 pN [36] have been reported for single dyneins. Moreover, in [36], the authors note that measurements in the cellular environment might represent an underestimate of the stall forces, because several factors can cause a motor to stop. In the case of kinesin, while the traditional results indicate stall forces in the range 6 pN–7 pN, new results indicate *F_s_*∼5 pN [18] *in vitro* and *F_s_*∼2.5 pN *in vivo* [18], [35]. In this paper, we show that our main results obtained for the symmetric system with F_s_∼6 pN regarding velocity distribution multimodality and reversion shapes for loose mechanical coupling are maintained when considering lower values of the stall force in the range 1–6 pN (Figures 6 and S8). Interestingly, we were able to reproduce the experimental results in *Xenopus* melanophores for values of the stall force in the range of the ones reported in living cells (F_s_>2 pN).

Finally, there is also a controversy on whether kinesins and dyneins participate in transport in a symmetric way or not. While studies in [10] suggest an asymmetric motor competition in *Dictyostelium* cells for which authors estimate that a number of dyneins between 4 and 8 with F_s_∼1.1 pN (measured *in vitro*) work against 1 or 2 kinesins of F_s_∼5 pN, other *in vivo* studies in different systems [18], [35], [36] suggest similar stall forces and similar number of motors for both species. Our simulations with symmetric and asymmetric configurations suggest that a model considering similar stall forces and similar number of motors for both species is in better accord with the results obtained for melanosomes than a very asymmetric one.

In a recent paper Kunwar *et al.* studied the transport of lipid droplets by kinesin and dynein in *Drosophila* embryos [18]. Their results suggest that besides mechanical tug-of-war, an additional level of regulation might be involved in the transport processes. Our studies indicate that relevant aspects of cargo transport in *Xenopus laevis* melanophores can be explained by only considering a tug-of-war model. However, we cannot discard the existence of additional regulation mechanisms.

Finally, although our studies focused on reproducing and interpreting previous experimental results for Xenopus laevis melanosomes transport driven by kinesin 2 and cytoplasmic dynein along microtubules, we believe that our results may be of interest to explain transport properties in a wide variety of biological systems.

## Supporting Information

Information S1 **Selection of pieces of processive motion towards a given direction.**
(DOC)

Information S2 **Approximate invariance of dynamical properties under parameter transformations.**
(DOC)

Information S3 **Fitting of multimodal velocity distributions.**
(DOC)

Figure S1 **Splitting of the velocity distribution by configurational states.** A) Velocity distribution for the parameter set in Table 1 of main text showing positive and negative velocity branches. Panels B to I show the contribution of the different pulling states. For simplicity, we do not include the contribution of the pulling state (0,0) which is almost vanished for this system.
(TIF)

Figure S2 **Split by pulling states of the velocity distribution of a system** ***N_f_ = N_b_*** **= 3.** Results correspond to the system with *N_f_ = N_b_* = 3 analyzed in Figure 3I of main text. For simplicity we show only the contributions of the most relevant states. See discussion in section Results of main text.
(TIF)

Figure S3 **Split by pulling states of the velocity distribution of a system with** ***N_f_ = N_b_*** **= 1.** The results correspond to the system with ***N_f_ = N_b_*** **= **1 analyzed in Figure 3I of the main text. Clearly, the dominant state is (1,0).
(TIF)

Figure S4 **Characteristic time of the slowing down process before reversions.** To determine the characteristic time of the reversion, we followed the same procedure as in [38], which is schematized here. Upper panel: time course of cargo position corresponding to a representative long-term reversion obtained with the parameters of the RS. The red curve represents the fitting of Eq. (2) to the data points just before the reversal. The initial of the slowing down segment was computed as the point where the position vs. time dependence deviates from linearity (green line). Lower panel: residuals of the fitting of Eq. (2) with A = 604 nm, *t_r_* = 750 ms and c = 53 nm.
(TIF)

Figure S5 **Reversions in systems with small stiffness.** Details of two wave shaped reversions obtained with the parameter set shown in Table 1 of main text (*k* = 0.02 pN/nm). The indications are as those for Figure 7. The insets in the top panels show the complete simulated trajectories with a red window indicating the long-term reversion considered.
(TIF)

Figure S6 **Reversions in systems with large stiffness.** Idem Figure S5 for two reversions obtained with the parameter set of Figure 3G with *k* = 0.3 pN/nm.
(TIF)

Figure S7 **High velocity segments.** Each panel shows a piece of trajectory obtained for different simulations using the parameters values displayed in Table 1. In each panel, the red mark indicates a 40 point-segment of large velocity included in the velocity distributions in Figure 3B. The insets show the dependence of the number of attached forward and backward motors during the large velocity segments. As can be seen, large segmental velocities are induced by detachment of opposing motors.
(TIF)

Figure S8 **Wave shaped reversions at low stall forces.** Panel A, *F_s_* = 4 pN, n_γ_ = 333 and *F_d_* = 2.4 pN. Panel B, *F_s_* = 2.7 pN, n_γ_ = 225 and *F_d_* = 1.7 pN. In both cases the values of *k* and ε are indicated in the panels while the remaining parameters are those displayed in Table 1.
(TIFF)
