# Supplementary material for: Transport Properties of Melanosomes along Microtubules Interpreted by a Tug-of-War Model with Loose Mechanical Coupling
Source: PLoS One. 2012 Aug 30;7(8):e43599. doi: 10.1371/journal.pone.0043599 (PMC3431353; doi:10.1371/journal.pone.0043599)
Supplement: Information S1 — Selection of pieces of processive motion towards a given direction. (DOC) [file pone.0043599.s001.doc]

**Supporting Information S1**

**Selection of pieces of processive motion towards a given direction**

In reference [8], the authors selected pieces of trajectories showing a clear trend towards a given direction (despite the small noise obtained from point to point) for times longer than 2 seconds. This procedure was performed by eye inspection and the selected segments were used for ulterior analysis. In this work, we automatized the detection of these periods in the numerically obtained trajectories. To do so, we performed a smoothing of each trajectory and then evaluated the difference between each pair of consecutive data points from the smoothed data. If a change of direction eventually occured, this difference changes its sign. The program then saves the data points where the inversion occurrs. These points determine the end of the different processive periods. Finally, the program selects the ones that lasted for more than two seconds.

Previously, we determined the degree of the smoothing procedure: if the smooth degree is too low (high), our algorithm will underestimate (overestimate) the periods with a global trend. Thus, we analyzed a few trajectories with different levels of smoothing and chose the one which resulted in trends similar to the ones that would have been chosen by eye inspection. The smoothing routines were run within the Matlab environment (The MathWorks, Inc.) and make use of the Neural Network Toolbox functions.
