# Supplementary material for: Transport Properties of Melanosomes along Microtubules Interpreted by a Tug-of-War Model with Loose Mechanical Coupling
Source: PLoS One. 2012 Aug 30;7(8):e43599. doi: 10.1371/journal.pone.0043599 (PMC3431353; doi:10.1371/journal.pone.0043599)
Supplement: Information S2 — Approximate invariance of dynamical properties under parameter transformations. (DOC) [file pone.0043599.s002.doc]

**Supporting Information S2**

**Approximate invariance of dynamical properties under parameter transformations**

Given a dynamical model with a large number of parameters it is always desirable to find a combination of parameter transformations that leaves the dynamics invariant. This reduces the effective dimensionality of the parameter space and, thus, facilitates the analysis [S1,S2]

Here we introduce a parameter transformation, which enables us to relate solutions of the model for different values of the stall force. For simplicity we discuss the symmetric model with equal parameters for forward and backward motors, although the same can be done with asymmetric parameter sets.

The model presented in this paper is found to be invariant under the following simultaneous changes of the parameters:

*F_s_*→λ *F_s_*,  *F_d_*→λ *F_d_*,  *k*→λ *k*,  γ→ λγ,  *T*→λ *T                          *(S3)*,   *

where λ is a positive number. This can be checked simply replacing the scaled parameters in the model equations (see Proof 1 below). From now on, we consider 0< λ<1. The cases λ >1 correspond to the inverse transformations.

However, the transformation S3 has a drawback when considering biological systems: the temperature is also re-scaled. This problem can be overcome if we consider the following transformation:

*F_s_*→λ *F_s_*,  *F_d_*→ β *F_d_*,  *k*→λ *k*,  γ→ λγ                                     (S2)

with constant T, and β ~λ^1/2^ (see Proof 2 below) This scaling of the parameters leads to a system whose dynamical properties are quite similar to those from the original one and it enables exploring more reasonably different parameters values. In particular, we will use S2 to analyze the behavior of the model for different values of the stall forces.

In a number of simulations at room temperature we have verified that, within the range 0.4< λ <1 –which represents stall forces in the range 2.5-6 pN-, the properties of the solutions for the transformed parameter set given by S2 are similar to those from the referential set (RS). In particular, the velocity distributions display peaks located at the same positions (see Figure S9). Moreover, the distributions of the scaled systems depend on the system parameters in the same way as those of the RS (see Figures 3 and 6 of the main text).

Our simulations with λ<0.4 -with Fs<2.5pN- show that the solutions for the transformed parameter sets begin to differ from the one obtained for the RS (see case λ=0.2  in figure S9).

**Figure S9: Velocity distributions for symmetric parameter sets scaled with the heuristic transformation given by S2.** The red curve corresponds to the referential parameter set (RS) indicated in Table I of main text. The black, green and blue curves represent the velocity distributions for symmetric parameter sets transformed from the RS using S2 with the values of λ indicated in the panel considering β=0.8, β=0.57 and β=0.447, respectively. The resulting values of the stall forces are indicated in the panel.

Proof 1. The easiest way to verify the invariance under transformation S3 is the following. First, note that the change *k*→λ *k* implies a change *f_i_* →λ *f_i_* (see equation 1 of the main text), and the same for the reaction (load) forces *L_i_*, which turn to be λ*L_i_*. On the other hand, scaling of γ and *T* transform the noise intensity into λ^2^γ*K_B_T,* which is equivalent to add a factor λ to the noise term in equation (2) and keep the noise intensity unchanged [S3]. Then, we replace *f_i_* and *L_i_* by λ*f_i_* and λ*L_i_*, respectively, and the parameters *F_s_, F_d_,* and γ by the scaled ones in equations (2), (3) and (4). The λ factors result cancelled and we get again equations (2), (3) and (4) unchanged, with no lambda parameters and unchanged noise intensity γ*K_B_T.*

Proof 2: The reason why transformation given by S2 works is the following. Rescaling *F_s_, F_d_, k* and γ with a factor λ  but keeping constant *T* is equivalent to the change T→T/ λ alone. From the mathematical point of view, this means that the scaled set represents a system with all the same parameters but stronger noise intensity, since the temperature is directly related to the noise term in the Langevin equation. The main effect of such stronger noise is to increase detachment, since fluctuations of load forces are expected to grow in a factor λ^−1/2^. Thus, increasing the detachment force in a factor of order λ^−1/2^ can solve the problem. Hence, we consider β ~ λ λ^−1/2^ =λ^1/2^. Note that the exact value β =λ^1/2^ is consistent with the composition of transformation S2 (since (λ_1_)^1/2^(λ_2_)^1/2^=(λ_1_ λ_2_)^1/2^ ) and also with the inversions considering λ>1.

**References**

[S1] Nicolis G. 1995. *Introduction to nonlinear science*. Cambridge University Press.

[S2] Kasprzak W, Lysik B, and Rybaczuk M. 1990. *Dimensional analysis in the identification of mathematical models.* World Scientific Publishing Co.Pte.Ltd

[S3] Van Kampen NG (1992) *Stochastic processes in physics and chemistry.* North-Holland., editor: Elsevier Science Publishers B.V.
