# Supplementary material for: Transport Properties of Melanosomes along Microtubules Interpreted by a Tug-of-War Model with Loose Mechanical Coupling
Source: PLoS One. 2012 Aug 30;7(8):e43599. doi: 10.1371/journal.pone.0043599 (PMC3431353; doi:10.1371/journal.pone.0043599)
Supplement: Information S3 — Fitting of multimodal velocity distributions. (DOC) [file pone.0043599.s003.doc]

**Supporting Information S3**

**Fitting of multimodal velocity distributions**

The segmental velocities distributions were fitted with the multimodal distribution considered in [8]:

$$N(v)=\sum_{n=1}^{4} A_{n} e^{-\frac{(v-nv_{0})^{2}}{2\sigma^{2}}} (S1)$$

Previously, the velocity histograms were normalized following the criterion used in [8]: setting the maximum of the distribution equal to 1.

The expression given by S1 represents the sum of four Gaussian distributions with different weights (*An*), each centered in a multiple of *v_o_* and with the same σ. *v* is the segmental velocity of the organelle. The values of the different *An*, *v_o_* and σ were fitted using the Curve Fitting Toolbox from Matlab.

The parameters obtained for the fits of the histograms obtained using Set 1 (Fig.8A) and Set 2 (Fig. 8B) and shown in figure 8.C, are the following:

Set 2: forward motion (solid red curve): A_2_=0.56, A_3_=0.26, A_4_=0.08

Set 2: backward motion (solid blue curve): A_2_=0.8, A_3_=0.35, A_4_=0.2

Set 1: forward motion (dotted red curve): A_2_=0.6, A_3_=0.24, A_4_=0.12

Set 1: backward motion (dotted blue curve): A_2_=0.65, A_3_=0.3, A_4_=0.16

In all the cases: v_0_=220 nm/s, σ=100 nm/s, A_1_=1

The errors of all the fitted parameters are less than 15%.
