# Supplementary figures and images for: Transport Properties of Melanosomes along Microtubules Interpreted by a Tug-of-War Model with Loose Mechanical Coupling
Source: PLoS One. 2012 Aug 30;7(8):e43599. doi: 10.1371/journal.pone.0043599 (PMC3431353; doi:10.1371/journal.pone.0043599)

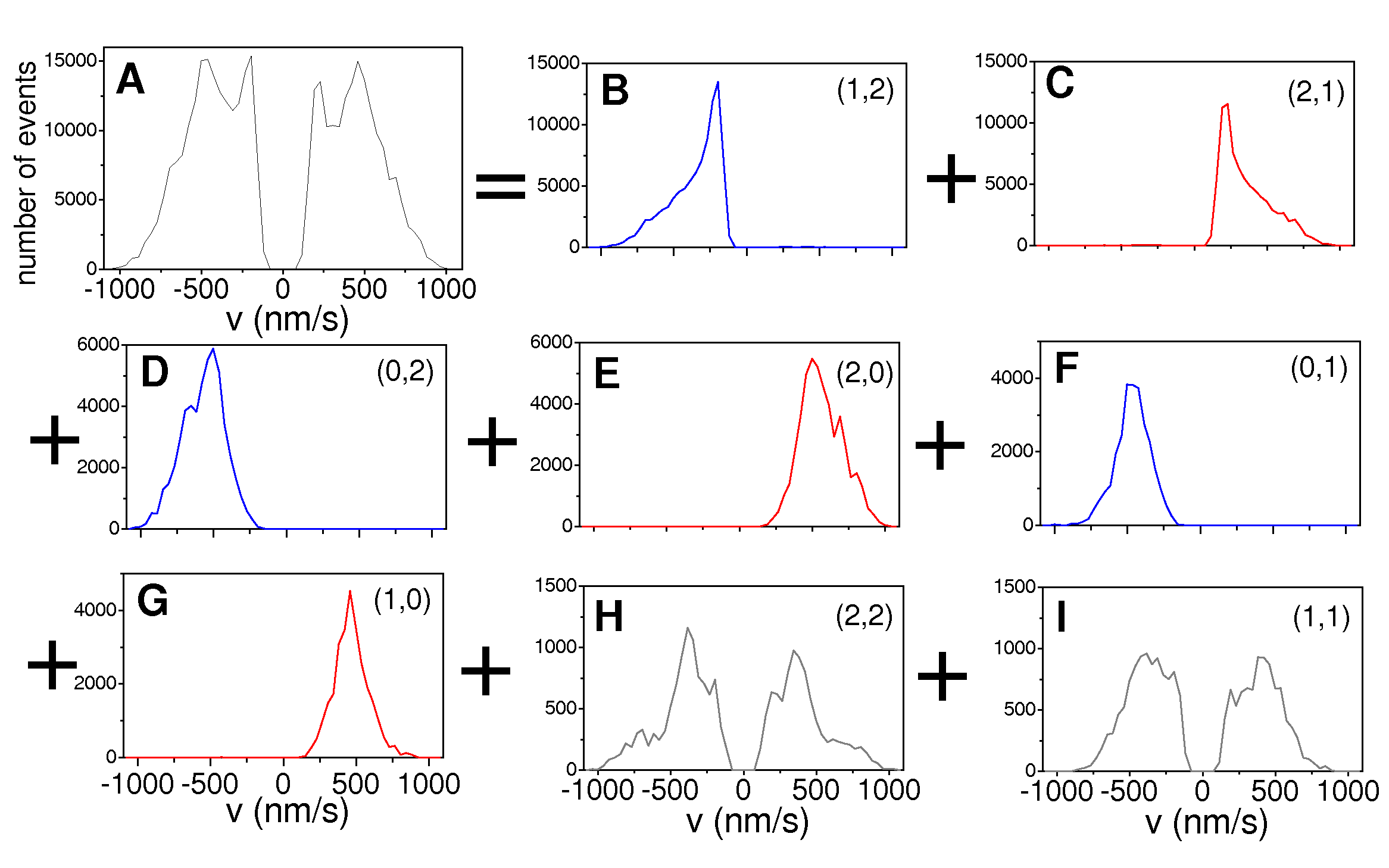

Supplement: Figure S1 — Splitting of the velocity distribution by configurational states. A) Velocity distribution for the parameter set in Table 1 of main text showing positive and negative velocity branches. Panels B to I show the contribution of the different pulling states. For simplicity, we do not include the contribution of the pulling state (0,0) which is almost vanished for this system. (TIF) [file pone.0043599.s004.tif]

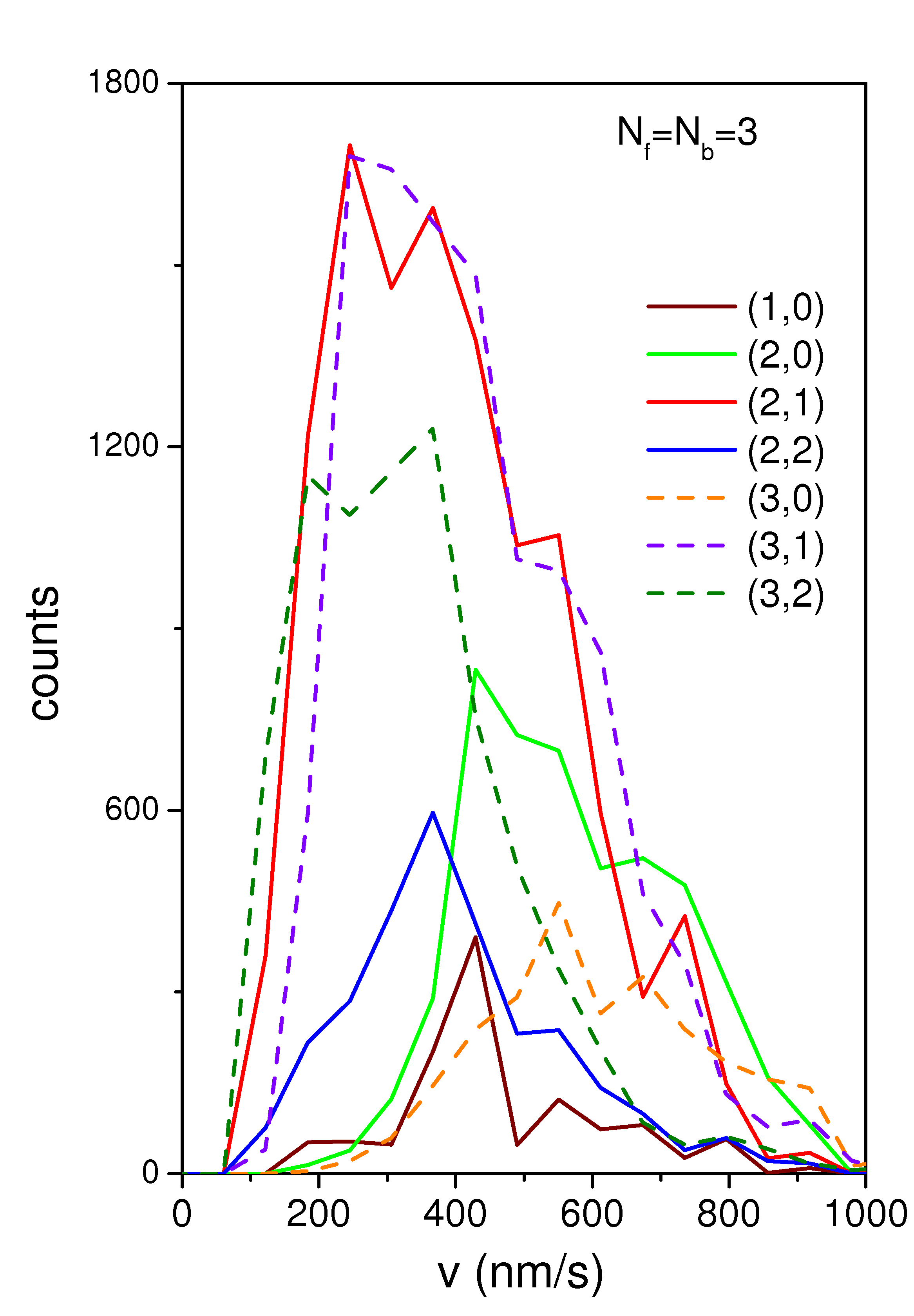

Supplement: Figure S2 — Split by pulling states of the velocity distribution of a system Nf = Nb = 3. Results correspond to the system with Nf = Nb = 3 analyzed in Figure 3I of main text. For simplicity we show only the contributions of the most relevant states. See discussion in section Results of main text. (TIF) [file pone.0043599.s005.tif]

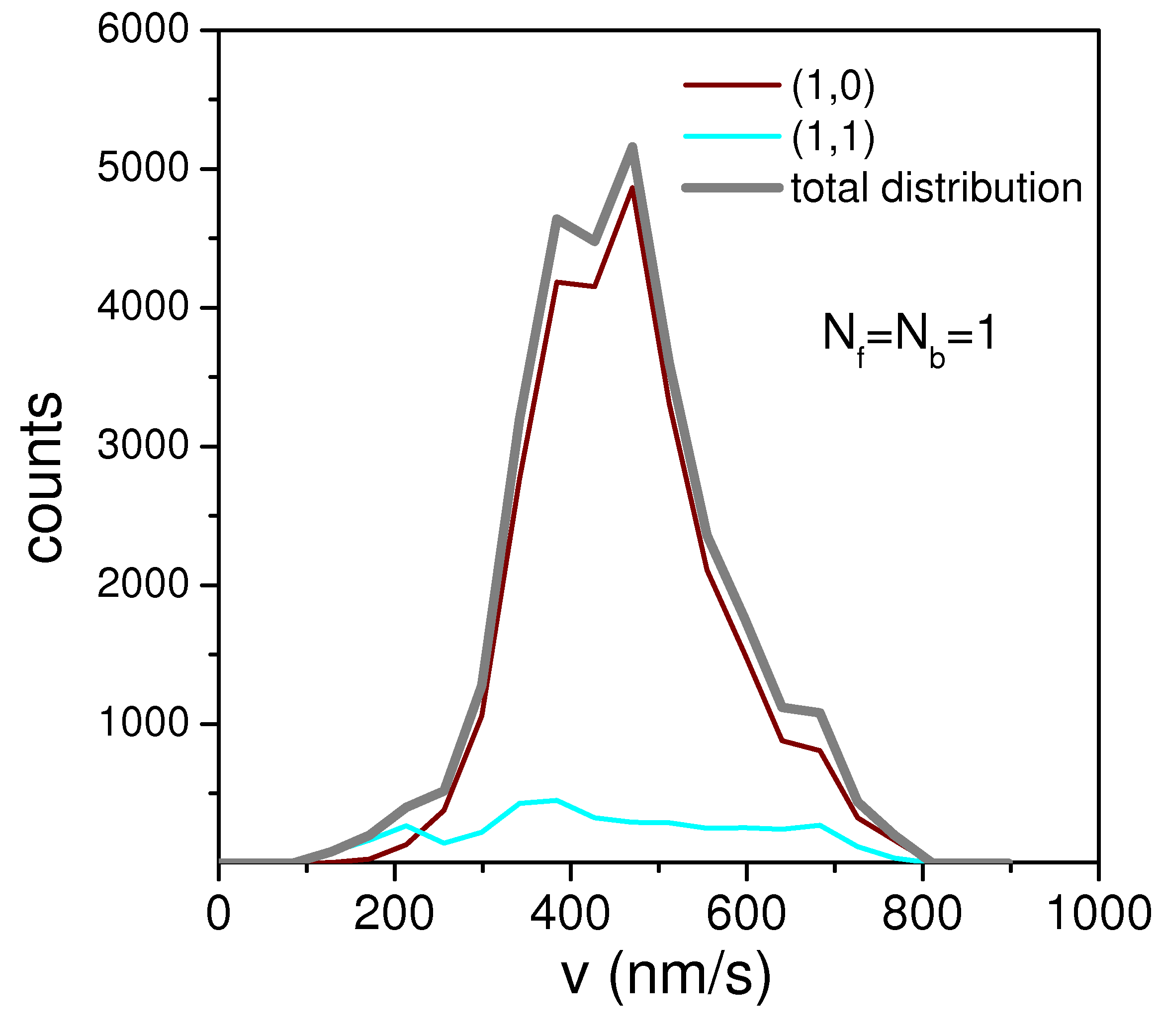

Supplement: Figure S3 — Split by pulling states of the velocity distribution of a system with Nf = Nb = 1. The results correspond to the system with Nf = Nb = 1 analyzed in Figure 3I of the main text. Clearly, the dominant state is (1,0). (TIF) [file pone.0043599.s006.tif]

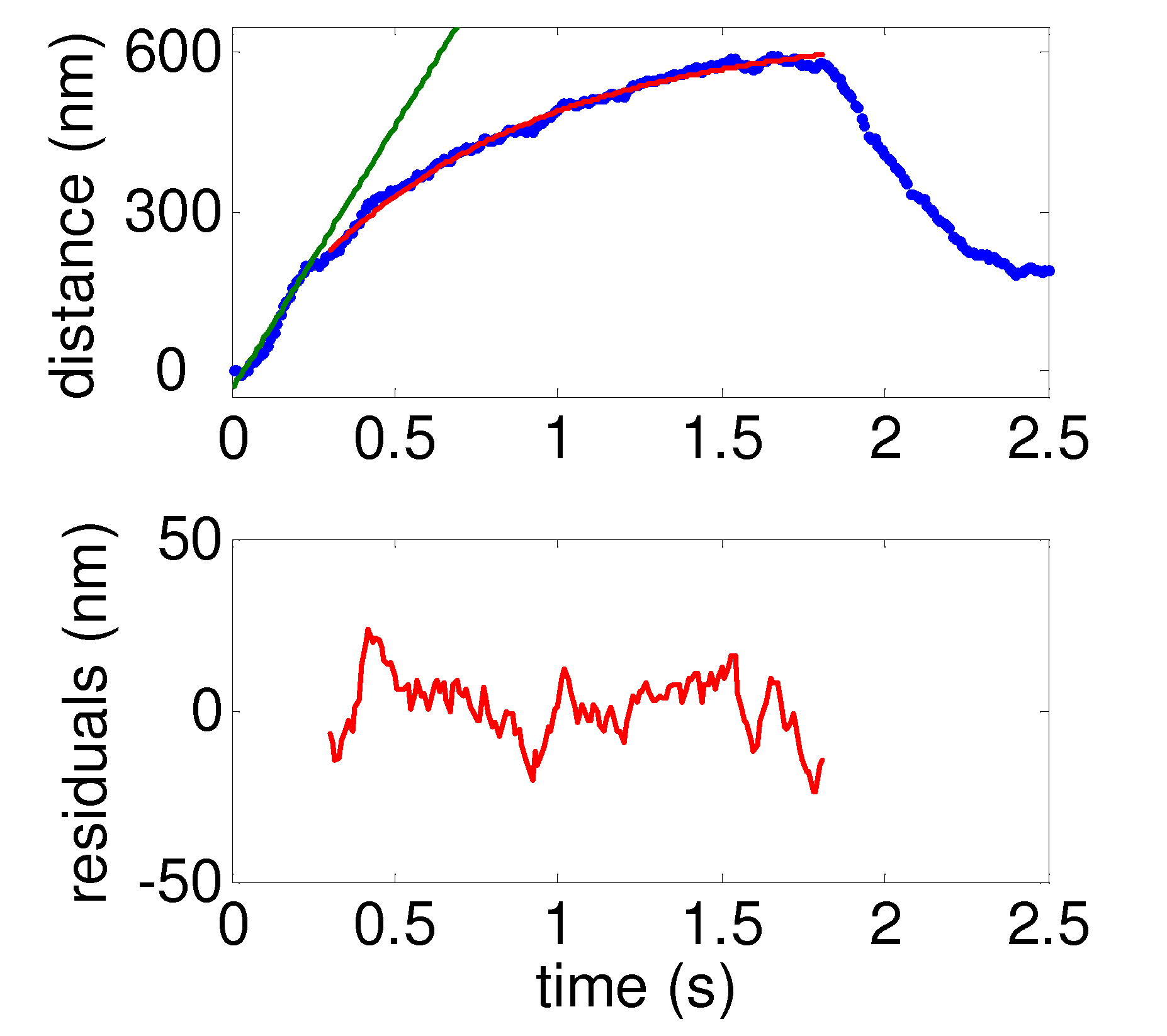

Supplement: Figure S4 — Characteristic time of the slowing down process before reversions. To determine the characteristic time of the reversion, we followed the same procedure as in [38], which is schematized here. Upper panel: time course of cargo position corresponding to a representative long-term reversion obtained with the parameters of the RS. The red curve represents the fitting of Eq. (2) to the data points just before the reversal. The initial of the slowing down segment was computed as the point where the position vs. time dependence deviates from linearity (green line). Lower panel: residuals of the fitting of Eq. (2) with A = 604 nm, tr = 750 ms and c = 53 nm. (TIF) [file pone.0043599.s007.tif]

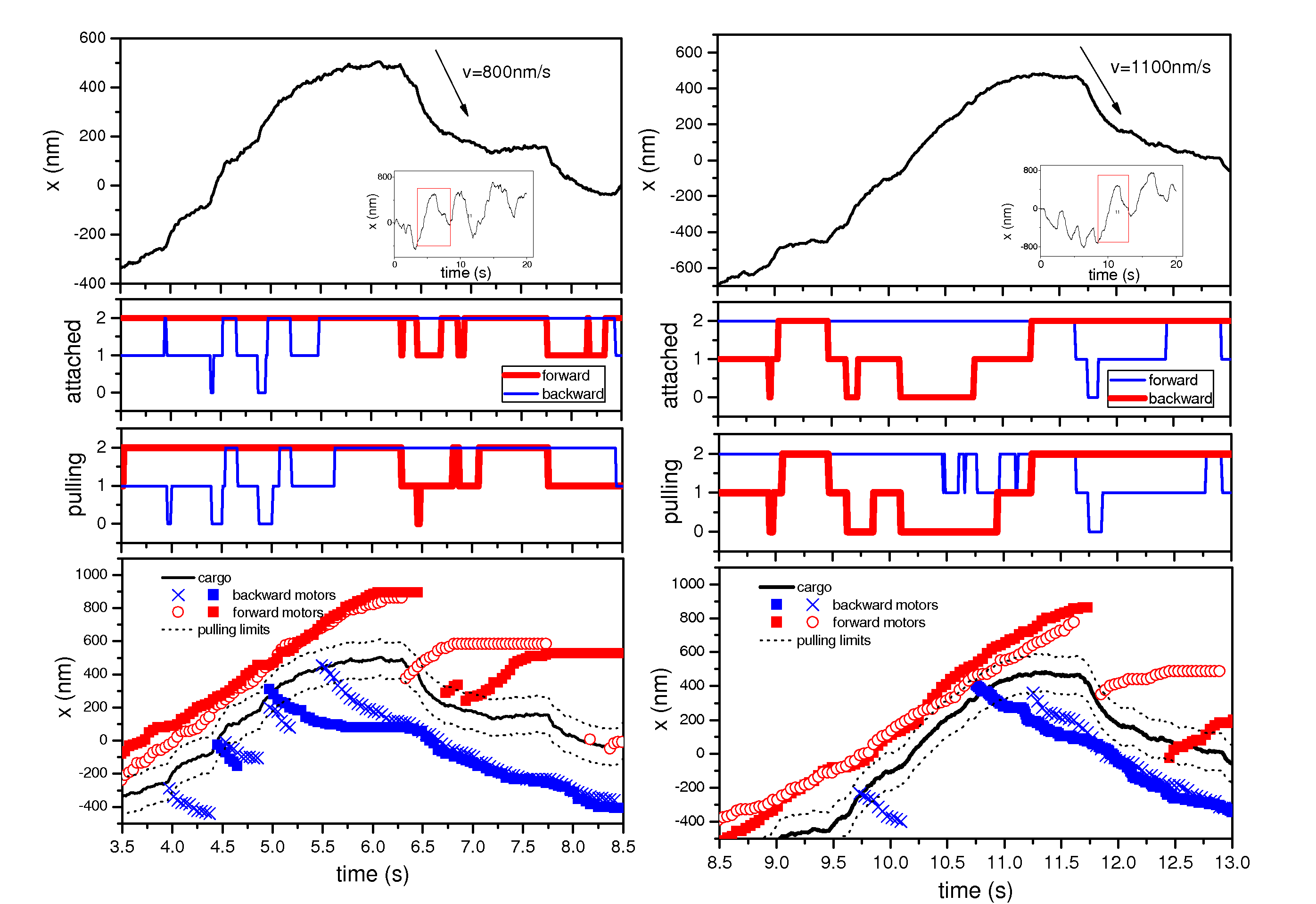

Supplement: Figure S5 — Reversions in systems with small stiffness. Details of two wave shaped reversions obtained with the parameter set shown in Table 1 of main text (k = 0.02 pN/nm). The indications are as those for Figure 7. The insets in the top panels show the complete simulated trajectories with a red window indicating the long-term reversion considered. (TIF) [file pone.0043599.s008.tif]

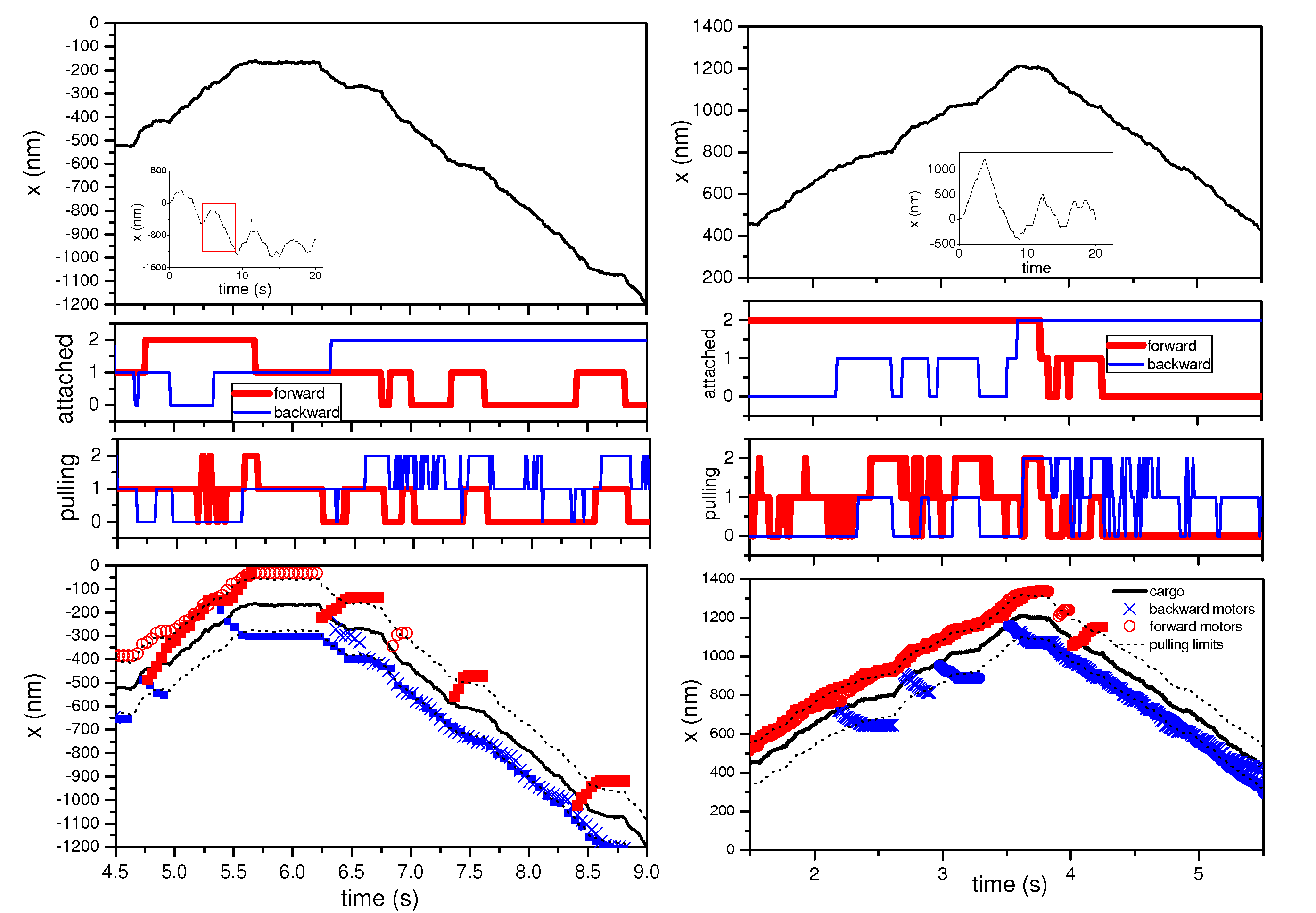

Supplement: Figure S6 — Reversions in systems with large stiffness. Idem Figure S5 for two reversions obtained with the parameter set of Figure 3G with k = 0.3 pN/nm. (TIF) [file pone.0043599.s009.tif]

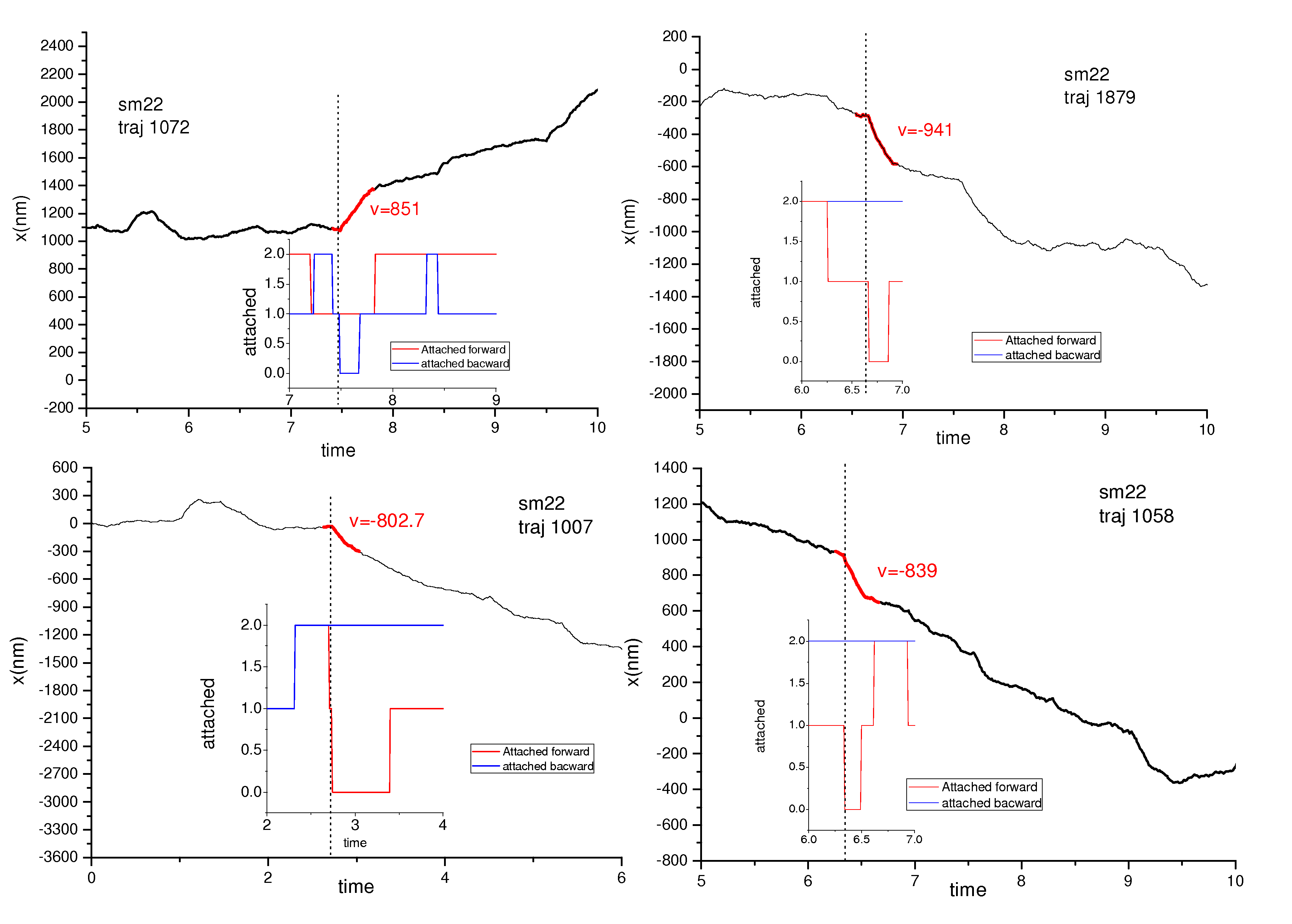

Supplement: Figure S7 — High velocity segments. Each panel shows a piece of trajectory obtained for different simulations using the parameters values displayed in Table 1. In each panel, the red mark indicates a 40 point-segment of large velocity included in the velocity distributions in Figure 3B. The insets show the dependence of the number of attached forward and backward motors during the large velocity segments. As can be seen, large segmental velocities are induced by detachment of opposing motors. (TIF) [file pone.0043599.s010.tif]

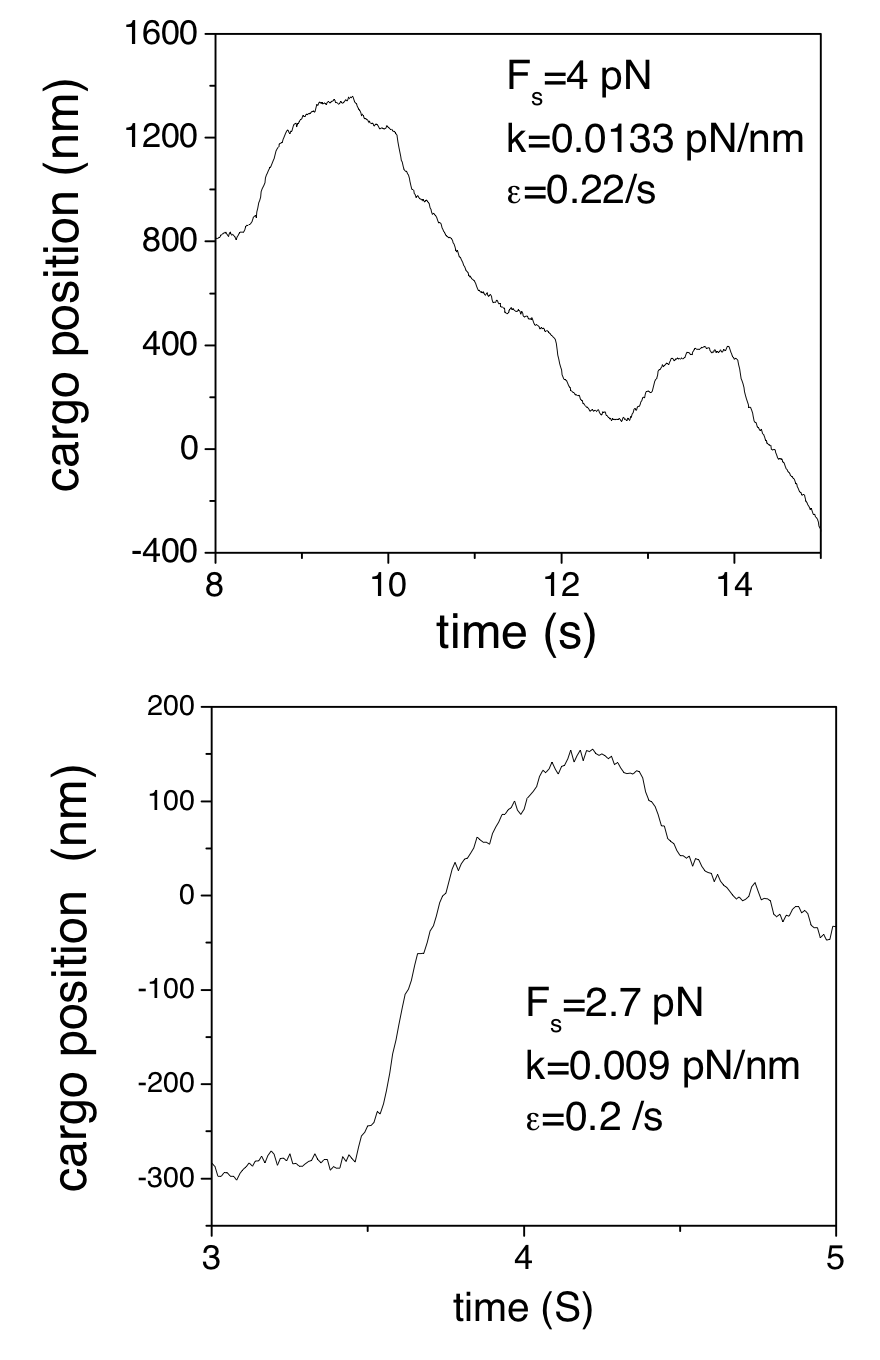

Supplement: Figure S8 — Wave shaped reversions at low stall forces. Panel A, Fs = 4 pN, nγ = 333 and Fd = 2.4 pN. Panel B, Fs = 2.7 pN, nγ = 225 and Fd = 1.7 pN. In both cases the values of k and ε are indicated in the panels while the remaining parameters are those displayed in Table 1. (TIFF) [file pone.0043599.s011.tiff]
